# Design and stability analysis of a new six-floater oscillating water column-based floating offshore wind turbine platform

**DOI:** 10.1038/s41598-024-65824-5

**Published:** 2024-07-13

**Authors:** Salvador Cayuela-Padilla, Fares M’zoughi, Izaskun Garrido, Aitor J. Garrido

**Affiliations:** Automatic Control Group—ACG, Institute of Research and Development of Processes—IIDP, Department of Automatic Control and Systems Engineering, Faculty of Engineering of Bilbao, University of the Basque Country—UPV/EHU, Po Rafael Moreno no3, 48013 Bilbao, Spain

**Keywords:** Mechanical engineering, Power stations, Devices for energy harvesting, Wind energy, Electrical and electronic engineering

## Abstract

The operational efficiency and lifespan of Floating Offshore Wind Turbines (FOWTs) are adversely impacted by the inherent platform motions and undesired vibrations induced by wind and wave loads. To effectively address these effects, the control of specific structural motions is of utmost importance, with platform pitch and yaw identified as the primary Degrees Of Freedom (DOF) that require attention. This study proposes a novel utilization of Oscillating Water Columns (OWCs) as a reliable and viable solution to mitigate platform pitch and yaw motions, thereby significantly enhancing the efficiency and reducing fatigue in wind turbines. This article aims to evaluate the impact resulting from integrating OWCs within each discrete floater of a Six-Floater platform. By considering different combinations of OWCs, a comprehensive analysis of the Response Amplitude Operators (RAOs) associated with pitch and yaw motions is presented. The primary objective is to identify the most efficient arrangements of OWCs and determine suitable combinations that effectively stabilize platform pitch and yaw motions. The empirical results substantiate that specific OWC configurations exhibit notable dampening effects on both pitch and yaw motions, particularly within specific wave frequency intervals. Consequently, it can be inferred that the integration and adequate operation of OWCs facilitate a substantial improvement in the stabilization of multi-floater platforms.

## Introduction

Floating offshore wind energy demand is increasing, being the European Union (EU) strategy to reach 60 GW by 2030 and 300 GW by 2050^1^. Current geopolitical events are strengthening the development of this technology to ease the energy independency of the EU and do not rely on imported fossil fuels, increasing the shear of wind power target from 40 to 45% by 2030 in the North Sea^2^. Moreover, this trend is not confined to the EU alone; it is spreading globally as countries recognize the potential of offshore wind energy. For instance, the Norwegian Government has set an ambitious target of achieving 30 GW of offshore wind capacity by 2040, which approximately matches the entire electrical consumption of Norway^3^. Such national targets underscore the growing recognition of floating offshore wind energy as a viable solution. To improve the efficiency of wind turbine power generation as well as maximizing the lifespan, it is crucial to ensure the stabilization of the floating platform. Among the various DOF associated with the platform, pitch motion has been identified as having the biggest substantial impact on turbine operation^4^. Extensive research has been conducted to explore mechanical solutions to enhance platform stabilization. One such passive approach involves the use of plates to dampen heave motion using hydrodynamic forces without the need for a control system^5^. An additional approach was formulated by Ding et al.^6^, whereby they implemented a Tuned Mass Damper (TMD) within the turbine nacelle to mitigate the aforementioned issue, leading to significant advancements in roll control.

Some suggestions are calling for the development of multifunctional platforms that mix various renewable energy sources. By harnessing energy from various sources simultaneously, this approach aims to enhance overall efficiency and reduce costs, thereby maximizing the utilization of the offshore platform. Among the most promising platforms designed for deep offshore waters are wind-wave energy platforms, as evidenced by the works of Hu et al.^7^, Sarmiento et al.^8^, Yu et al.^9^, and Gao et al.^10^. It has been recommended to employ Wave Energy Converters (WECs) in conjunction with a FOWT, which has shown favorable results. For instance, Kluger et al.^11^ investigated the implementation of a spar-based FOWT known as OC3-Hywind in conjunction with a wave energy converter array. Moreover, Ma et al.^12^ examined the impact of typhoon conditions on the aerodynamic performance of OC3-Hywind. Finally, Slocum et al.^13^ explored the effects of incorporating both outer and inner heaving wave energy converters within the same floating system. Another example was presented by Kamarlouei et al.^14^, who demonstrated that the adoption of a wave energy converter array can lessen the system's oscillations in the heave and pitch modes. Recently, Khatibani and Ketabdari^15^ conducted an investigation focusing on the dynamics and power absorption capabilities of two wave energy converters implemented within a hybrid monopile wind turbine.

The study of the feasibility and effectiveness of OWC devices as WECs for extracting energy from marine structures has gained significant attention within the academics. For example, Zheng et al.^16^ conducted a theoretical analysis with the objective of optimizing the size of WECs that contributed to the broader understanding of WECs and their potential applications independently of the ocean structure where they are installed. Wang et al.^17^ studied the hydrodynamic response of combined FOWT-WECs integrated systems and conducted experimental modeling and tank testing to validate their results. Similarly, Sarmiento et al.^8^ explored the combination of a 5 MW Floating Offshore Wind Turbine equipped with three OWCs, providing insights into the performance and potential advantages of this configuration. Another field of OWC WECs application is floating breakwaters, as studied by Howe et al.^18^, where they focused on the dual objectives of wave attenuation and energy extraction, highlighting the multifunctional capabilities of OWC devices.

Recent studies have focused on exploring the application of OWC devices as stabilizers that mitigate the response of FOWTs when subjected to the influence of incoming waves. Zhu et al.^19^ have conducted an experimental study integrating OWCs in a semisubmersible FOWT reduce the motion of the platform. M’zoughi et al.^20,21^ integrated two OWCs into a barge platform in front and behind a 5 MW National Renewable Energy Laboratory (NREL) wind turbine to study the reduction effects on vibration caused by heading waves by integrating the hydrodynamic forces induced by the OWCs. In a similar line, Aboutalebi et al.^22^ have studied the performance a barge-type FOWT platform equipped with four OWCs, showing significant reductions in oscillations, indicative of the potential of OWC integration in enhancing the stability and efficiency of the FOWT system.

Platform pitching and yawing motions induce disturbances in the wind flow field, resulting in aerodynamic effects that impact the overall performance of the wind turbine^23^. While pitch motion is the main parameter that influences the turbine efficiency, the analysis of yaw motion is equally important. The pitch and yaw motions of the platform produce a non-uniform wind flow in the rotor disk of the turbine, creating a skewed airflow with rapid, localized changes at the blade tips. These changes may lead to vortices that significantly affect the flow around the rotor disk, especially when coupled with those platform rotational motions, enlarging these unsteady aerodynamic effects. When the platform is yawing half of the wind turbine rotor disk is pointing upstream the incoming wind flow, moving forwards and facing higher winds and thus higher loads, while the other half is downstream, moving backwards. This has an effect on the power generation and its control, as well as the overall structural fatigue. In the case of yaw motion, it is necessary to assess how the platform's response affects the wind turbine. This response depends mainly on the floater hydrodynamics and especially in the mooring arrangement, requiring the latter a thorough analysis beyond the scope of this study.

**Figure 1:**
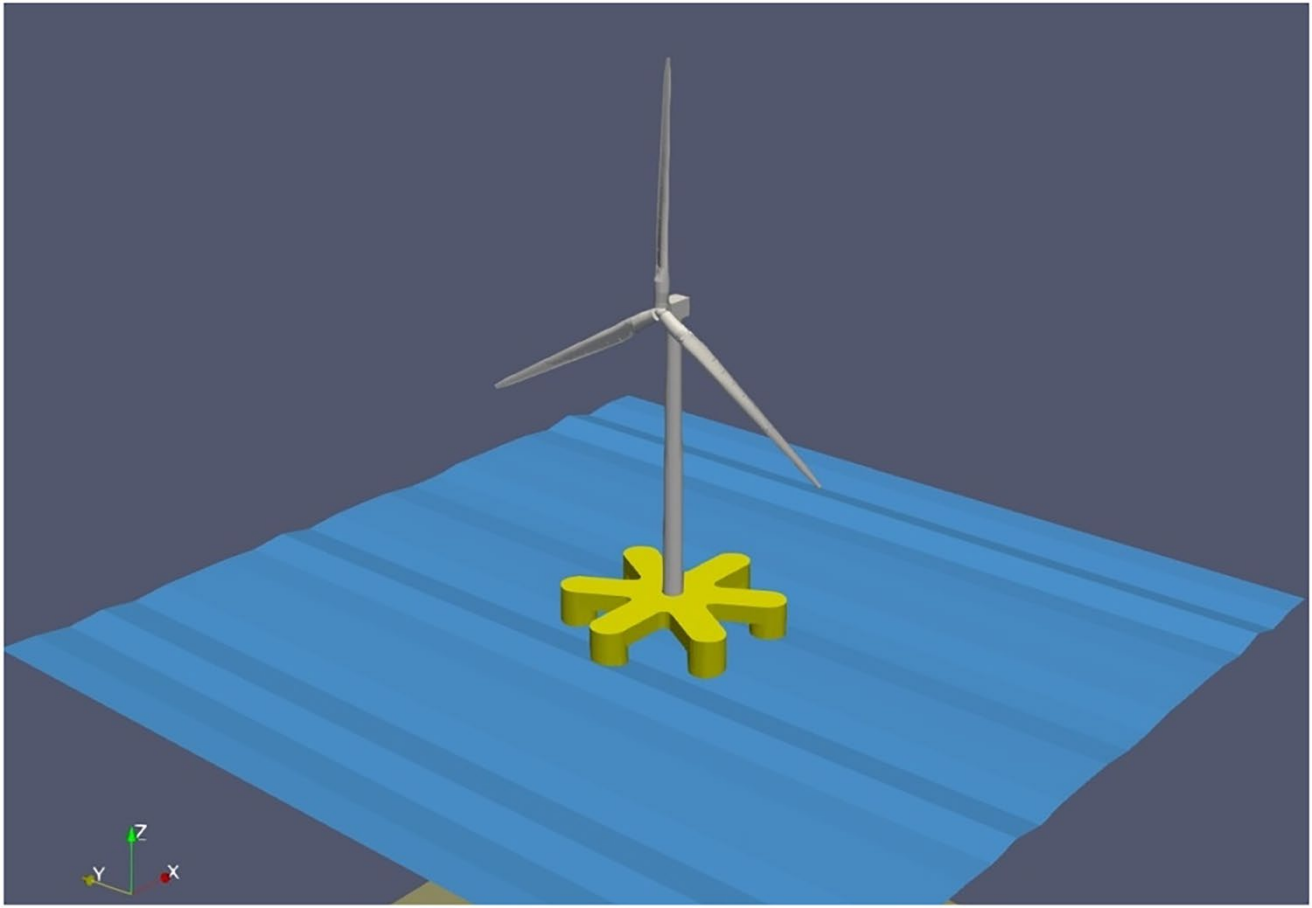
The proposed hybrid six-floater Oscillating Water Column-based Floating Offshore Wind Turbine Platform (6OWC-FOWT) concept with 5 MW wind turbine.

A novel hybrid Six Floater Oscillating Water Column-based Floating Offshore Wind Turbine Platform (6OWC-FOWT) with 5 MW wind turbine from NREL is analyzed in this work, as illustrated in Fig. 1. The main objective is to analyze the 6OWC-FOWT floater platform to ascertain the optimal arrangement of OWCs to effectively reduce both pitch and yaw motions. The results show significant dampening of the motions in both degrees of freedom (DOFs) within specific frequency intervals, highlighting the potential of certain OWCs' locations in achieving the desired reduction.

The remainder of the paper is organized as follows: Section “Design of the proposed hybrid six-floater oscillating water column-based floating offshore wind turbine platform concept” provides a comprehensive description of the design of the hybrid 6OWC-FOWT featuring a 5 MW wind turbine from NREL. Additionally, the details of the FAST model wind turbine employed in the study are outlined. In Section “Theoretical background and modeling”, a theoretical background of the FOWT, OWC and Wells turbine were introduced to tackle the mathematical modeling of the entire 6OWC-FOWT system. Section “Analysis of wave interactions”, focuses on the wave interaction of the floaters, whereby coefficients of the hydrodynamic matrixes obtained using WAMIT are employed to analyze the response of the floater. Section “Assessment of platform response”, introduces the chosen criterion to evaluate the performance of the new structure namely the RAO. The section elaborates on the procedure to obtain the RAOs, starting from calculation settings and preliminary processes, and ending with data post-processing to obtain the transfer function (RAO). In Section “Simulation results”, a simulation test is conducted and the response results are presented. Section “Discussion”, debates the results of the proposed 6OWC-FOWT floater concept. Finally, Section “Conclusions” ends the paper with some concluding remarks.

### Design of the proposed hybrid six-floater oscillating water column-based floating offshore wind turbine platform concept

The proposed hybrid six-floater Oscillating Water Column-based Floating Offshore Wind Turbine Platform (6OWC-FOWT) concept is developed using the CAD software MultiSurf v8.9, which facilitates the design of complex geometries and has a seamless integration with WAMIT.

FAST solver couples the aerodynamic, hydrodynamic and (servo) control induced loads, considering the structural elastic dynamic response of the offshore wind turbine in the time domain, together with the hydrodynamic and maintaining wave-induced response of the floating platform, with the aid of WAMIT software.

**Figure 2:**
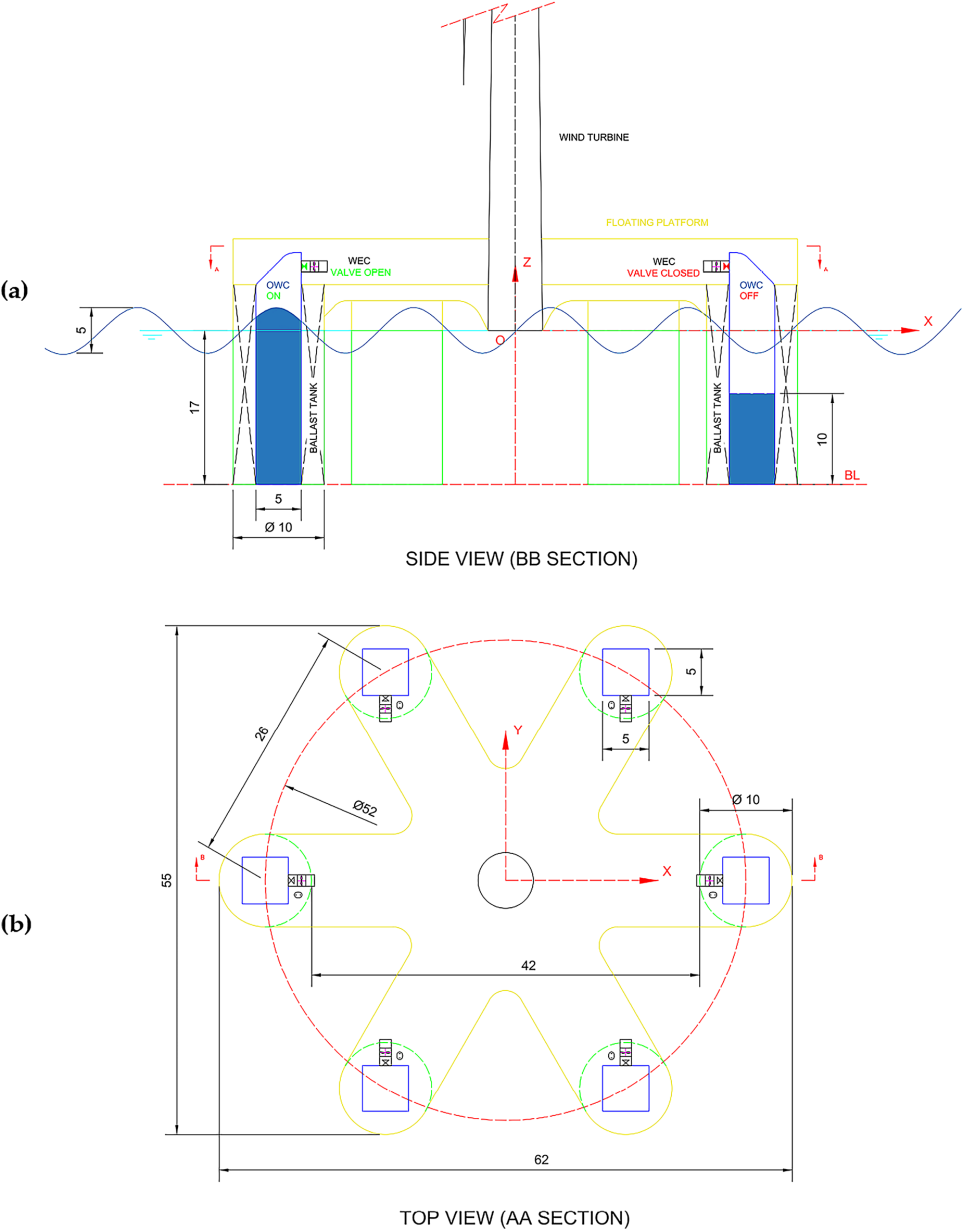
General arrangement of Six–Floater platform submerged body (measurements in m).

The 6OWC-FOWT platform consists of a 5 MW wind turbine mounted on a set of six cylindrical floaters of 5 m radius. Figure 2 (a) provides an illustration of the platform, depicting the inclusion of an OWC chamber within each floater, equipped with a controlled valve at the top. The six floaters are located around the wind turbine forming a circular arrangement of 52 m in diameter, at 60 deg from each other, and 26 m apart, as illustrated in Fig. 2b.

The primary objective of this study is to investigate and compare the dynamic behavior of the six-floater platform, specifically the 6OWC-FOWT, under wave excitations with and without the presence of Oscillating Water Columns (OWCs). The integration of the six OWCs within the 6OWC-FOWT platform aims to achieve optimal stabilization of the platform across various sea conditions. Consequently, the study seeks to identify the most suitable combination of OWC operation (valve open) based on the analysis of platform responses for different associations of OWCs, taking into consideration the given wave frequency.

Table 1 provides key specifications and details of the 6OWC-FOWT floater platform, outlining its main characteristics and relevant parameters that will be considered throughout the analysis and evaluation of the platform's performance.

**Table 1:** Six-Floater platform particulars, including OWCs. The platform is ballasted to keep a depth of 17 m, in any OWC operation condition.

Dimension/particulars	Data
Platform; length, beam, depth	62 m, 55 m, 17 m
Floater diameter	10 m
Distance between floaters’ centers	26 m
Draft (full load)	17 m
Displacement six-floater, with no OWC in operation	6511 ${m}^{3}$
Displacement six-floater, with six OWCs in operation	5461 ${m}^{3}$
Capture Chamber width	5 m
Vertical center of gravity (VCG)	− 9.0 m
Radii of gyration, X axis (Roll)	20.93 m
Radii of gyration, Y axis (Pitch)	20.92 m
Radii of gyration, Z axis (Yaw)	26.35 m
Water depth	150 m

**Figure 3:**
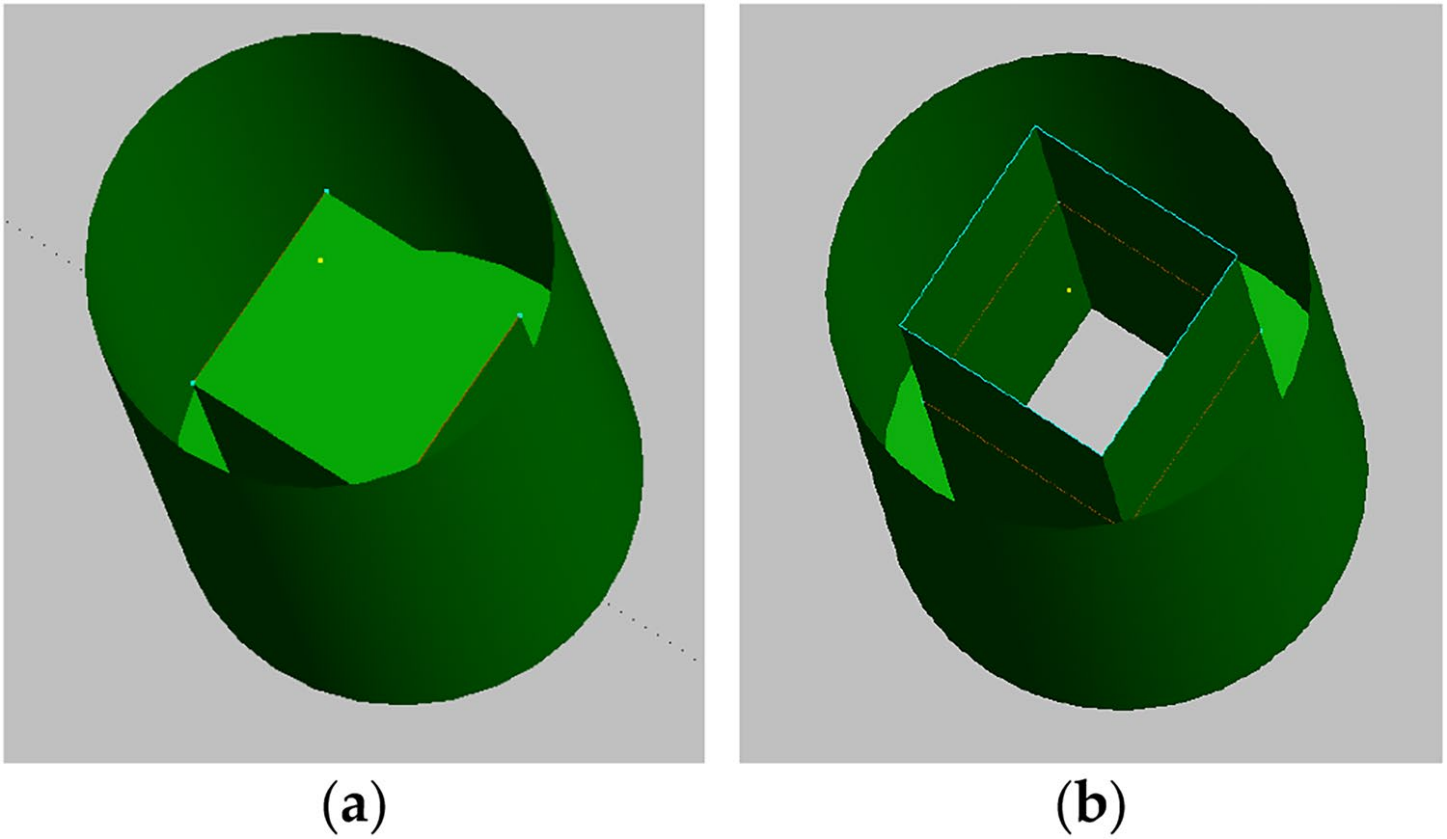
(**a**) Model of cylindrical floater and OWC closed chamber; (**b**) The OWC chamber is extended up to the water level to simulate the OWC in operation.

**Figure 4:**
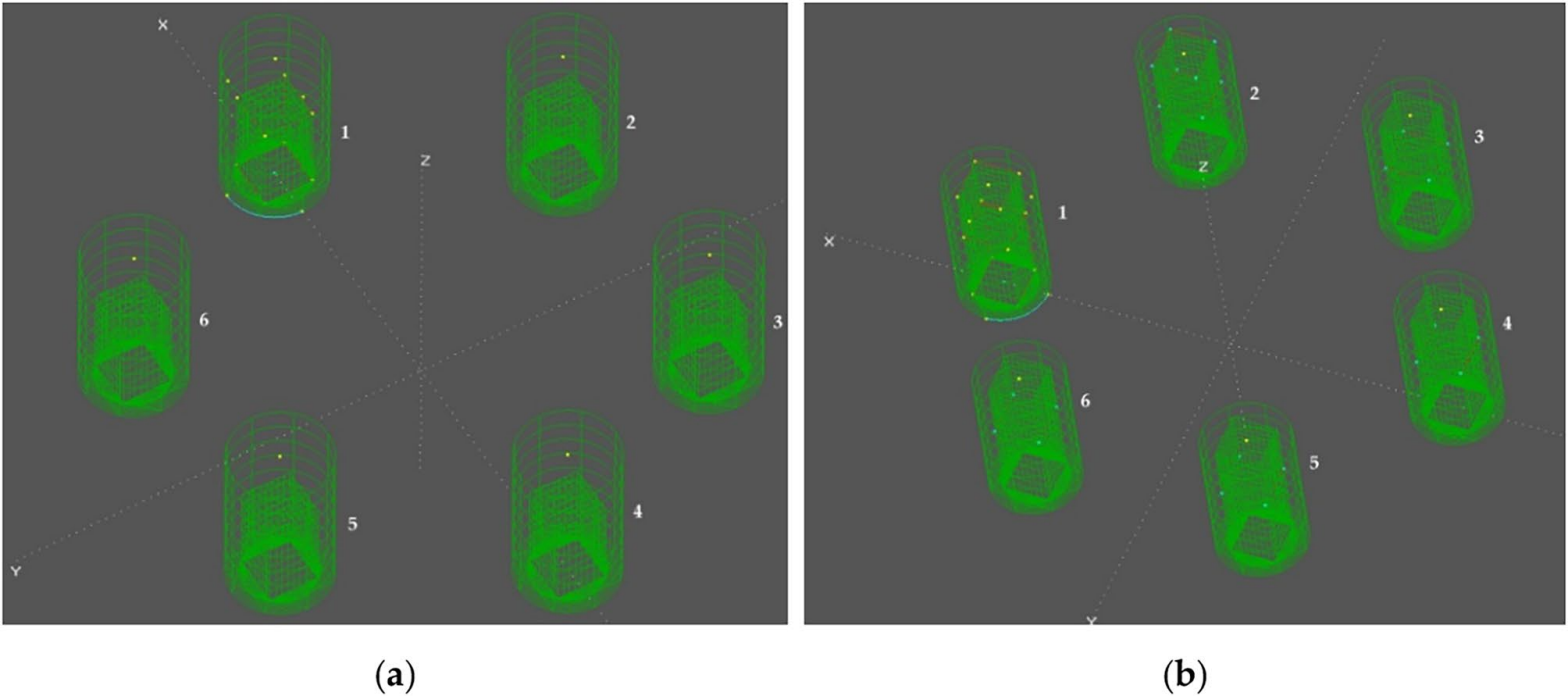
Floaters numbering. (**a**) Wireframe view of the platform model with all OWC chambers closed; (**b**) Wireframe view of the model with all OWC in operation.

The OWC in operation has been modeled with the chamber up to the sea water level, and the OWC out of operation mode has been modeled as a closed chamber of 10 m depth from Base Line (BL), as shown in Fig. 2 (side view), and Fig. 3 (general view). In Fig. 4, the floaters are labeled consecutively in a clockwise manner, starting with the floater positioned along the positive X-axis, denoted as "1."

The proposed modeling approach allows for the simulation of the platform's behavior throughout the operational range, starting from the ballast condition with the six OWCs chambers closed (displacement of 6511 ${m}^{3}$) until the scenario where all OWCs chambers are opened (displacement of 5461 ${m}^{3}$). This modeling approach of the closed chamber neglects the air compressibility, so that it warrants further investigation, and its implications will be addressed in the subsequent Discussion section.

The radii of gyration may vary for different OWC combinations, and this parameter is calculated in each simulation. The radii of gyration data that appears in Table 1 corresponds to the scenario in which all OWC chambers are opened.

This article considers the NREL 5 MW baseline Wind Turbine, as described in the work by Jonkman et al.^24^. The key specifications and characteristics of this wind turbine are presented in Table 2. The wind turbine properties used in our floating platform solution are similar for the fixed-bottom application.

**Table 2:** Main particulars of the NREL 5 M W Baseline Wind Turbine.

Dimension/particulars	Data
Rating	5 MW
Hub height	90 m
Location of overall center of mass	64.0 m
Rotor diameter	126 m
Number of blades	3
Rated speed of rotor	12.1 rpm
Cut-in, rated, cut-out wind speed	3 m/s, 11.4 m/s, 25 m/s
Mass of blades	53,220 kg
Mass of hub	56,780 kg
Mass of nacelle	240,000 kg
Mass of tower	347,460 kg

The mooring design used in the 6OWC-FOWT platform is equivalent to the layout used in the ITI Energy Barge^25^, consisting of eight catenary mooring lines with a 4 × 2 arrangement, being the fairleads for attachment of the lines located in floaters number 2, 3, 5 and 6. The main properties of the mooring lines are summarized in Table 3.

**Table 3:** Main properties of the mooring lines (ITI Energy Barge).

Dimension/particulars	Data
Water depth	150 m
Unstretched line length	773.8 m
Neutral line length resting on seabed	250 m
Line diameter	0.0809 m
Line mass density	130.4 kg/m
Line extensional stiffness	589,000,000 N

### Theoretical background and modeling

**Figure 5:**
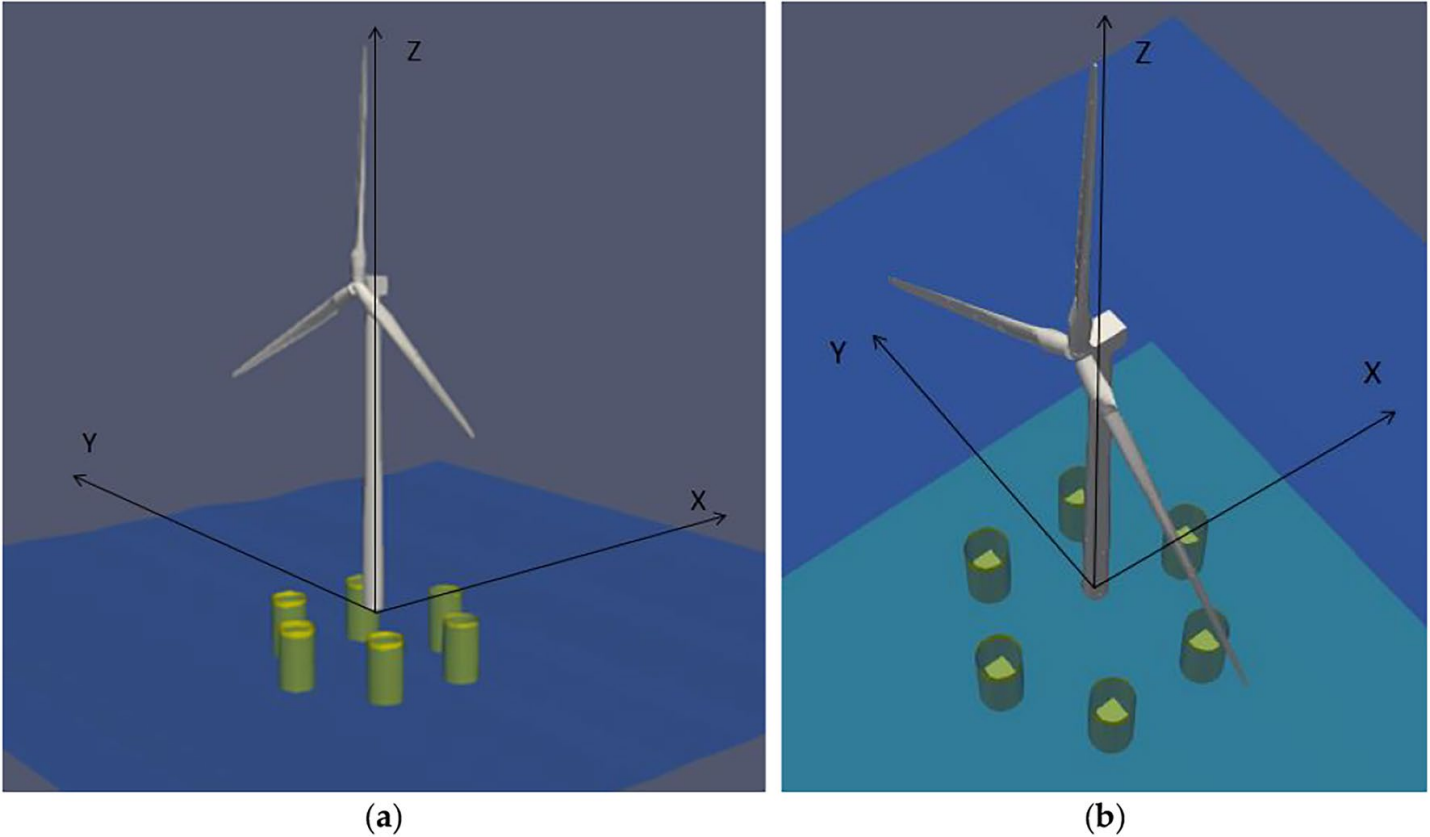
Coordinate system used for the 6OWC-FOWT floater concept. (**a**) General view (only submerged part modeled for the platform); (**b**) Top view showing the integrated OWCs in every floater (non operation case with closed valves).

For the OWC study, the dynamics of ocean waves are modeled using the Airy linear theory^26^ which can be described as:

1 $$z\left( {x,t} \right) = A \cdot \sin \left( {\omega t - kx} \right) = \frac{H}{2} \cdot \sin (\omega t - kx)$$

2 $$k=\frac{2\pi }{\lambda }$$

where $H$ is the wave height (distance between the through and crest of the wave), $\omega$ is the wave frequency and $k$ is the wave number. In this article, the coordinate system is defined such that the plane Z = 0 corresponds to the plane of the free surface, also known as the still water level (SWL). The Z-axis is oriented positively upwards, as illustrated in Fig. 5.

According to Jonkman ^25^, the time—domain equation that describes the motion of the whole system has the following form:

3 $${M}_{ij}(q,u,t){\ddot{q}}_{j}={f}_{i}(q,\dot{q},u,t)$$

where ${M}_{ij}$ is the component (i,j) of the inertia mass matrix, which is function of q, the number of DOFs; u, the number of control inputs and the time $t$. The second derivative in time of *j*th DOF is ${\ddot{q}}_{j}$. Eventually, ${f}_{i}$ is the component of the force associated to the *i*th DOF.

The equation of the system in frequency domain can be written as follows:

4 $$M\left(\omega \right)\ddot{q}+B\left(\omega \right)\dot{q}+Cq=f\left(\omega \right)+{f}_{OWC}\left(\omega \right)$$

where $M$ is the inertia matrix, $B$ is the damping matrix and $C$ is the restoring matrix of the whole system, $f\left(\omega \right)$ is the exciting force of the waves, and ${f}_{OWC}\left(\omega \right)$ is the response force of the OWCs.

The inertia matrix of the whole system (FOWT + OWC) is next:

5 $$M\left(\omega \right)={A}_{Added\_Mass}\left(\omega \right)+{M}_{Platform}+{M}_{Wind\_Turbine}$$

where ${M}_{Platform}$ is the inertia matrix of the platform, ${M}_{Wind\_Turbine}$ is the mass matrix of the wind turbine set, and ${A}_{Added\_Mass}\left(\omega \right)$ is the added mass matrix of the FOWT.

The platform mass matrix is defined as follows^27^:

6 $${M}_{Platform}=\left[\begin{array}{cccccc}m& 0& 0& 0& m{z}_{g}& -m{y}_{g}\\ 0& m& 0& -m{z}_{g}& 0& m{x}_{g}\\ 0& 0& m& m{y}_{g}& -m{x}_{g}& 0\\ 0& -m{z}_{g}& m{y}_{g}& {I}_{11}& {I}_{12}& {I}_{13}\\ m{z}_{g}& 0& -m{x}_{g}& {I}_{21}& {I}_{22}& {I}_{23}\\ -m{y}_{g}& m{x}_{g}& 0& {I}_{31}& {I}_{32}& {I}_{33}\end{array}\right]$$

where $$m$$ is the platform mass and ($${x}_{g}, {y}_{g},{z}_{g})$$ are the coordinates of the center of gravity. The moments of inertia $${I}_{ij}$$ are defined by $${I}_{ij}=m{r}_{ij}\left|{r}_{ij}\right|$$, where $${r}_{ij}$$ is the radii of gyration.

The mass matrix of the wind turbine ${M}_{Wind\_Turbine}$ is calculated by FAST. The calculation comprises the 6 × 6 matrix for a rigid body motion plus the DOFs of an elastic wind turbine, as blade bending, tower bending, rotor rotation and drive train torsion^28^. The added mass matrix ${A}_{Added\_Mass}\left(\omega \right)$ is calculated by WAMIT.

The restoring matrix $C$ is formed by the corresponding restoring matrix of the floater ${C}_{Floater}$, calculated by WAMIT, plus the stiffening matrix of the mooring lines ${C}_{Mooring}$:

7 $$C={C}_{Floater}+{C}_{Mooring}$$

The damping matrix $B\left(\omega \right)$ is composed of the damping matrix of the floaters associated to the hydrodynamic radiation ${B}_{Floater}\left(\omega \right)$, the damping matrix of the hydrodynamic viscous drag ${B}_{Viscous}$, the damping matrix of the elastic wind turbine ${B}_{Wind\_Turbine}$ and the damping effect of the OWC acting as a WEC (energy harvesting), ${B}_{OWC}$:

8 $$B\left(\omega \right)={B}_{Floater}\left(\omega \right)+{B}_{Viscous}+{B}_{Wind\_Turbine}+{B}_{OWC}$$

The effect of the OWC ${f}_{OWC}\left(\omega \right)$, can be modeled as a mechanical cylinder^29^:

9 $${f}_{OWC}\left(\omega \right)=-p\left(\omega \right)S$$

where $p$ is the pressure drop and $S$ is the interior free surface.

Considering the air as an ideal gas and the compression and decompression process isentropic, the air density is defined by:

10 $$\rho = \rho _{0} \left( {\frac{p}{{p_{0} }}} \right)^{{{\raise0.7ex\hbox{$1$} \!\mathord{\left/ {\vphantom {1 \gamma }}\right.\kern-\nulldelimiterspace} \!\lower0.7ex\hbox{$\gamma $}}}}$$

where ${p}_{0}$ and ${\rho }_{0}$ are the initial pressure and density (OWC out of operation) and $\gamma$ is the heat capacity ratio of air. If Eq. (10) is linearized:

11 $$\dot{\rho }=\frac{{\rho }_{0}}{{\gamma p}_{0}}\dot{p}$$

The linearized mass flow of an axial-flow turbine can be defined as:

12 $$\dot{m}=\frac{d\left(\rho V\right)}{dt}=\frac{{\rho }_{0}}{{\gamma p}_{0}}\dot{p}{V}_{0}+{\rho }_{0}\dot{\text{V}}$$

where $V$ is the volume of the air cylinder.

An axial-flow Wells turbine of turbine diameter *D* and rotational speed *N* is defined by following^30^:

13 $$\psi =K\phi$$

where $\psi$, the pressure coefficient is defined by:

14 $$\psi =\frac{p}{{\rho }_{0}{N}^{2}{D}^{2}}$$

and $\phi$, the flow coefficient is defined by:

15 $$\phi =\frac{\dot{m}}{{\rho }_{0}N{D}^{3}}$$

Considering the pressure drop proportional to the flow rate,

16 $${\psi }_{c}={K}_{c}{\phi }_{c}$$

where ${\psi }_{c}$, the pressure coefficient is defined by:

17 $${\psi }_{c}=\frac{p}{{\rho }_{0}gH}$$

being $H$ the wave height, and ${\phi }_{c}$, the flow coefficient is defined by:

18 $${\phi }_{c}=\frac{2\pi \dot{m}}{{\rho }_{0}\omega SH}$$

Combining (16), (17) and (18), the mass flow is defined by:

19 $$\dot{m}=\frac{\omega S}{2\pi g{K}_{c}}p$$

where $${K}_{c}$$ is a constant value that depends on the wave steepness $${\raise0.7ex\hbox{$H$} \!\mathord{\left/ {\vphantom {H L}}\right.\kern-\nulldelimiterspace} \!\lower0.7ex\hbox{$L$}}$$.

Combining (12) and (19) and transforming the formula into the complex field, we obtain the value of the pressure complex amplitude $\widehat{p}$:

20 $$\widehat{p}=i\omega \frac{\Gamma }{\omega \left[1+{\left(\varepsilon \Gamma \right)}^{2}\right]}\frac{1}{S}\widehat{V}-{\omega }^{2}\frac{\varepsilon {\Gamma }^{2}}{\omega \left[1+{\left(\varepsilon \Gamma \right)}^{2}\right]}\frac{1}{S}\widehat{V}$$

being $\widehat{V}$ the complex amplitude of volume and $\Gamma$ and $\varepsilon$ constant values defined as:

21 $$\Gamma =2\pi g{\rho }_{0}{K}_{c}$$

22 $$\varepsilon =\frac{{V}_{0}}{{\gamma p}_{0}S}$$

Combining (9) and (20), and considering $\widehat{V}=S\widehat{q}$, it can be obtained the damping and stiffness coefficients as:

23 $${f}_{OWC}\left(\omega \right)=-i\omega \frac{\Gamma }{\omega \left[1+{\left(\varepsilon \Gamma \right)}^{2}\right]}\widehat{q}+{\omega }^{2}\frac{\varepsilon {\Gamma }^{2}}{\omega \left[1+{\left(\varepsilon \Gamma \right)}^{2}\right]}\widehat{q}$$

24 $${B}_{OWC}\left(\omega \right)=\frac{\Gamma }{\omega \left[1+{\left(\varepsilon \Gamma \right)}^{2}\right]}$$

25 $${C}_{OWC}\left(\omega \right)=\frac{\varepsilon {\Gamma }^{2}}{\omega \left[1+{\left(\varepsilon \Gamma \right)}^{2}\right]}$$

This way the Eq. (4) can be written as:

26 $$M\left(\omega \right)\ddot{q}+\left[B\left(\omega \right)+{B}_{OWC}\left(\omega \right)\right]\dot{q}+\left[C+{C}_{OWC}\left(\omega \right)\right]q=f\left(\omega \right)$$

The exciting force of the waves $f\left(\omega \right)$ is calculated by WAMIT, which uses the boundary integral equation method to obtain the velocity potential and fluid pressure, so viscous forces in the fluid are not considered.

The set of equations from (9) to (25) are part of a general, accurate modeling framework aiming to represent the effect of the PTO as an external force. This allows its integration into WAMIT, by means of the generalized modes of the system, using a damping matrix of an external action subject to an equivalent viscous damping, which will require further research and development, including experimental support. Nevertheless, the simplified model focusing on the effect of the air chambers, as conducted in our current work, is a valid approach that remains included in the general modeling framework. This approach yields the structural response of our 6OWC-FOWT platform in the different combinations of OWC operation modes.

### Analysis of wave interactions

The matrixes ${A}_{Added\_Mass}\left(\omega \right)$, ${B}_{Floater}\left(\omega \right)$, ${C}_{Floater}$ and $f\left(\omega \right)$ are calculated by WAMIT, a linear frequency domain panel code, that analyses the response of floating and submerged bodies. It is based on linear and second order potential theory. The velocity potential and fluid pressure is calculated by the boundary integral equation method (BIEM). Further details about the coordinate system employed may be found at the beginning of the Section “Theoretical background and modeling”.

According to WAMIT user manual^31^, the volume $\forall$ is calculated with next expression, considering Gauss divergence theorem:

27 $$\forall =-{\iint }_{{S}_{b}}{n}_{1}xdS=-{\iint }_{{S}_{b}}{n}_{2}ydS=-{\iint }_{{S}_{b}}{n}_{3}zdS$$

where $n=\left({n}_{1}, {n}_{2},{n}_{3}\right)$ is unit vector normal to the body surface that points out to the fluid field.

The coordinates of center of buoyancy are given by:

28 $${x}_{b}=-\frac{1}{2\forall }{\iint }_{{S}_{b}}{n}_{1}{x}^{2}dS$$

29 $${y}_{b}=-\frac{1}{2\forall }{\iint }_{{S}_{b}}{n}_{2}{y}^{2}dS$$

30 $${z}_{b}=-\frac{1}{2\forall }{\iint }_{{S}_{b}}{n}_{3}{z}^{2}dS$$

The coefficients of the hydrostatic restoring matrix ${C}_{Floater}$ are next:

$$C\left(\text{3,3}\right)=\rho g{\iint }_{{S}_{b}}{n}_{3}dS$$

$$C\left(\text{3,4}\right)=\rho g{\iint }_{{S}_{b}}y{n}_{3}dS$$

$$C\left(\text{3,5}\right)=-\rho g{\iint }_{{S}_{b}}x{n}_{3}dS$$

$$C\left(\text{4,4}\right)=\rho g{\iint }_{{S}_{b}}{y}^{2}{n}_{3}dS+\rho g\forall {z}_{b}-mg{z}_{g}$$

$$C\left(\text{4,5}\right)=-\rho g{\iint }_{{S}_{b}}xy{n}_{3}dS$$

$$C\left(\text{4,6}\right)=-\rho g\forall {x}_{b}+mg{x}_{g}$$

$$C\left(\text{5,5}\right)=\rho g{\iint }_{{S}_{b}}{x}^{2}{n}_{3}dS+\rho g\forall {z}_{b}-mg{z}_{g}$$

31 $$C\left(\text{5,6}\right)=-\rho g\forall {y}_{b}+mg{y}_{g}$$

For the rest of values $i,j$, the coefficients are $C\left(i,j\right)=0$. In general, $C\left(i,j\right)=C\left(j,i\right)$ for all values of $i,j$, except $C\left(\text{6,4}\right)=C\left(\text{6,5}\right)=$ 0. The coordinates of the center of gravity of the submerged body are $\left({x}_{g},{y}_{g},{z}_{g}\right)$, and $m$ the mass of the body.

The added mass and damping coefficients are next:

$${A}_{ij}-\frac{i}{ \omega }{B}_{ij}=\rho {\iint }_{{S}_{b}}{n}_{i}{\varphi }_{j}dS$$

$${\overline{A} }_{ij}=\frac{{A}_{ij}}{ \rho {L}^{k}}$$

32 $${\overline{B} }_{ij}=\frac{{B}_{ij}}{ \rho {L}^{k}\omega }$$

where $k=3$ when $\left(i=1, 2, 3\right)$, $\left(j=1, 2, 3\right)$; $k=4$ when $\left(i=1, 2, 3\right)$, $\left(j=4, 5, 6\right)$ or $\left(i=4, 5, 6\right)$, $\left(j=1, 2, 3\right)$; $k=5$ when $\left(i=4, 5, 6\right)$, $\left(j=4, 5, 6\right)$. ${\overline{A} }_{ij}$ and ${\overline{B} }_{ij}$ are the normalized values of the added mass and damping coefficients, being $L$ the length scale.

### Assessment of platform response

The methodology employed for determining the response of the floating offshore platform consists of several distinct processes, as outlined in the work by Ramachandran et al.^28^. The initial step involves defining the geometric model using MultiSurf (v8.9), a computer-aided design software. Subsequently, WAMIT (v7) utilizes this model to compute the coefficients of various hydrodynamic matrices and the force excitation induced by incoming waves in the selected incident wave directions. These computed data are then used as inputs for the FAST (v8) software, which simulates the coupled response of the platform with the NREL 5 MW Baseline Wind Turbine, following the methodology described by Jonkman et al.^24^. This response in the time series is further transformed into the frequency domain to study the Frequency Response Function (FRF) of the platform. This is also referred to as Response Amplitude Operator (RAO), which is the ratio between the input, represented by the auto-spectral density of the surface wave elevation, with the output, represented by the cross-spectral density of the system's response:

33 $$RAO=\frac{{S}_{xy}\left(\omega \right)}{{S}_{xx}\left(\omega \right)}$$

being ${S}_{xy}\left(\omega \right)$ the cross spectral density and ${S}_{xx}\left(\omega \right)$ the auto spectral density of the wave elevation input $x\left(t\right)$ and system response output $y\left(t\right)$ signals.

The spectral densities ${S}_{xy}\left(\omega \right)$ and ${S}_{xx}\left(\omega \right)$ are averaged out the results from a minimum number of simulations, which are computed as the product of the Fast Fourier Transform (FFT) of the input or output signal by the complex conjugate of the input signal:

34 $${S}_{xy}\left(\omega \right)=\frac{1}{s}\sum_{i=1}^{s}{Y}_{i}\cdot {X}_{i}^{*}$$

35 $${S}_{xx}\left(\omega \right)=\frac{1}{s}{\sum }_{i=1}^{s}{X}_{i}\cdot {X}_{i}^{*}$$

where $s$ is the number of simulations ($s=6$ in our case), ${Y}_{i}$ is the FFT of the output signal $y\left(t\right)$ at the simulation $i$, ${X}_{i}$ is the FFT of the input signal $x\left(t\right)$ at simulation $i$, and ${X}_{i}^{*}$ is the complex conjugate of ${X}_{i}.$

The RAOs obtained through the FAST simulation are derived from random white-noise spectrum wave inputs in the time domain. To ensure accuracy and reliability, six separate simulations are conducted, each employing different seed numbers for random white noise wave excitation, and then averaged to obtain a representative RAO. In order to mitigate any transient effects, the initial 2000 s of each 8000 s duration simulation are discarded.

In order to analyze and compare between two or more systems simulations the integral of the Power Spectral Density (PSD) is used as a suitable metric for data analysis^32^. The PSD is firstly calculated from the RAO function and later integrated in a certain frequency interval.

### Simulation results

The performance evaluation of the novel 6OWC-FOWT floater concept has been carried out using the RAO and the PSD integral. The RAOs are obtained by subjecting the system to random noise excitations, which is a widely adopted approach in practical applications due to its ability to provide more accurate results compared to using periodic signals. By analyzing and evaluating the RAOs, the motions of the platforms can be assessed. The RAOs for different modes of the system enable us to make informed decisions regarding the platform's performance under varying sea states. These RAOs serve as valuable indicators of the platform's dynamic response and aid in determining which configuration exhibits superior performance characteristics. However, although RAOs are a useful tool to assess the response of some number of systems (such as different OWC combinations compared to the 000 000 baseline model), it is necessary to use another metrics, as the integral of the PSD. This metric enables the analysis of great amount of data, allowing for comparison between all OWC combinations in different load conditions. In the present work, due to the large amount of data to be analyzed, a hybrid approach using RAOs and integral of PSDs has been employed. The integral of PSDs provides a rough estimation of the system's behavior across the entire frequency range, while RAOs pinpoint specific frequency intervals of interest for particular effects. For ease of reference, all the results for the target platform motions under different wind load condition have been gathered in Tables 5 and 6.

#### Platform structural response

In this study, the procedure outlined in Section “Assessment of Platform Response” is adopted to plot the RAOs and calculate de PSD integrals. The numerical conditions for generating the RAOs are as follows. The simulation time for each computation is set to 8000 s, with an initial transient period of 2000 s discarded to mitigate any transient effects. Random white-noise wave inputs with a significant wave height of 2 m are utilized to ensure adherence to the linear wave theory. Six computations of the spectral densities ${S}_{xy}\left(\omega \right)$ and ${S}_{xx}\left(\omega \right)$ are performed, and the results are averaged. Next, PSD integrals are derived from the RAO function and integrated over a specified frequency interval.

Moreover, certain environmental conditions are assumed for the simulations. The incoming waves are assumed to have a heading direction of zero degrees (positive X-axis), Two wind cases are considered for the simulations: near-rated wind speed of 12 m/s, and below cut-out wind speed of 24 m/s. A normal turbulence model (according to the International Electrotechnical Commission turbulence type classification) corresponding to the standard number IEC 61,400-1-ED3, with IEC turbulence characteristic A is applied to the air conditions. Although the wind control and aerodynamic loads are not part of the main scope of the study, they are involved in all case-studies, as pitch control is active in all simulations. Indeed, the aerodynamics loads are considered in every simulation in order to compute the dynamics of the system -e.g., to calculate the bending moment at the base of the tower. Nonlinearities arising from the flexible tower, hydrodynamic loads, and mooring lines are taken into consideration. However, the nacelle-yaw control servo mechanism is not applied in the simulations.

**Figure 6:**
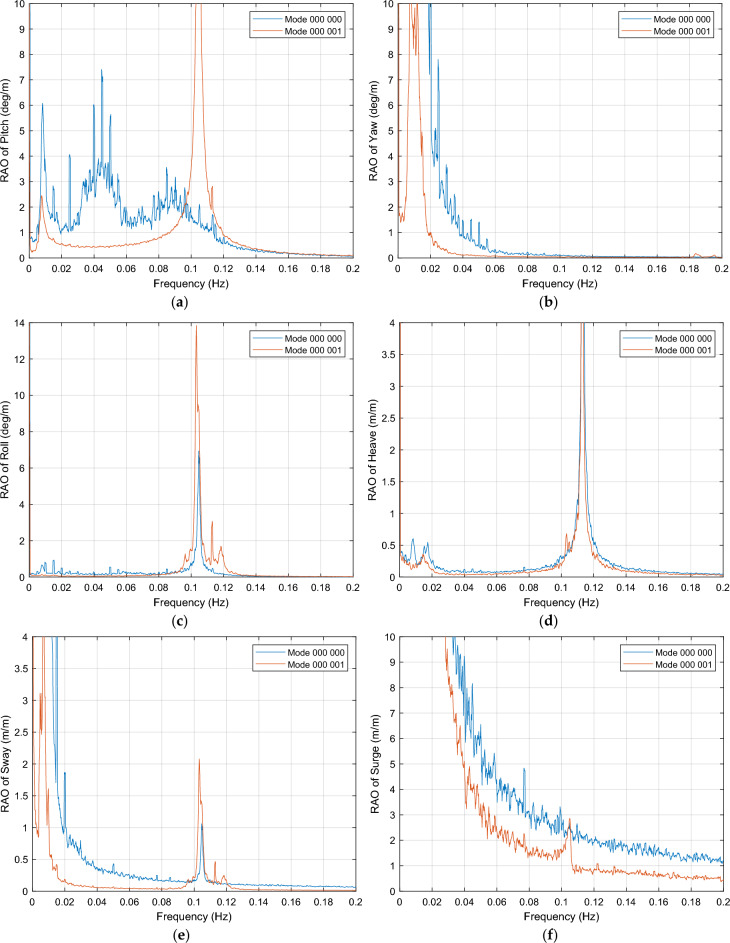
RAO of the 6OWC-FOWT floater structure with all OWC valves closed (operation mode 000 000) versus when valve of OWC in position 6 is opened (operation mode 000 001), in near-rated wind speed condition. (**a**) Pitch motion; (**b**) Yaw motion; (**c**) Roll motion; (**d**) Heave motion; (**e**) Sway motion; (**f**) Surge motion.

The RAOs for the six DOFs in a near-rated wind speed condition of 12 m/s of the novel 6OWC-FOWT floater concept with frequency range from 0 Hz to 0.2 are presented in Fig. 6a–f. The blue curves represent the RAOs when all OWC valves are closed (operation mode 000 000), while the red curves represent the RAOs when the valve of OWC in position 6 is opened (operation mode 000 001). By comparing these two sets of curves, the effects of opening the OWC valve in position 6 on the platform's response can be observed and analyzed.

The analysis of the RAO results reveals significant reductions in almost all platform motions across a wide range of frequencies, indicating the overall efficiency of the 6OWC-FOWT floater concept in various wave periods. However, it is observed that around the resonance frequency of 0.105 Hz, for pitch, roll, and sway the motions responses have increased, as well as the heave motion. Moreover, the responses of motions other than heave deviate from their respective resonance peaks, allowing for wave energy harvesting within this frequency range without compromising the efficiency of wind energy harnessing.

To identify the resonances occurring at frequencies slightly above 0.1 Hz for the operation modes 000 001, 100 000 and 000 000, the resonance frequencies and damping coefficients for each DOF are obtained by means of free-decay calculations plus simulations with no wind to assess the wave resonance intervals. Table 4 gathers this information.

**Table 4:** Resonance periods and damping characteristics of selected OWC operation modes.

OWC Mode	Pitch ζ (%)	Pitch T (s)	Yaw ζ (%)	Yaw T (s)	Roll ζ (%)	Roll T (s)	Heave ζ (%)	Heave T (s)	Sway ζ (%)	Sway T (s)	Surge ζ (%)	Surge T (s)
000 000	4.5 ± 0.2	9.66 ± 0.02	6.79 ± 0.01	82.65 ± 0.68	0.5 ± 0.2	9.58 ± 0.02	0.02 ± 0.02	8.82 ± 0.07	0.16 ± 0.02	9.57 ± 0.01	0.78 ± 0.04	10.93 ± 0.77
000 001	4.39 ± 0.13	9.88 ± 0.02	6.02 ± 0.04	109.11 ± 1.83	0.55 ± 0.19	9.71 ± 0.01	0.05 ± 0.04	8.85 ± 0.06	4.74 ± 0.10	9.72 ± 0.01	6.28 ± 0.15	10.25 ± 0.17
100 000	3.92 ± 0.18	9.74 ± 0.14	6.80 ± 0.21	15.21 ± 6.78	0.85 ± 0.11	9.57 ± 0.04	0.15 ± 0.05	8.88 ± 0.06	0.15 ± 0.04	9.60 ± 0.04	0.93 ± 0.12	10.32 ± 0.47

**Figure 7:**
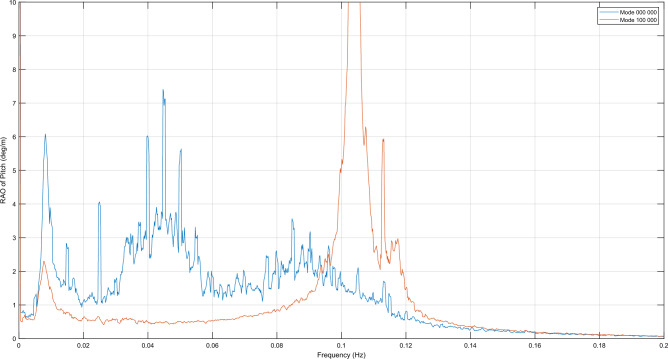
Pitch RAO in near-rated wind speed condition of the platform with all OWC WECs control valves closed (operation mode 000 000) versus Pitch RAO of the platform when valve of OWC in position 1 is opened (operation mode 100 000).

Based on the natural periods of the DOFs of operation mode 000 001 it can be inferred that the 0.1 Hz resonance in pitch, roll, heave and sway is due to OWC resonance. Additionally, OWC operation mode 100 000, which has a similar effect in pitch, as depicted in Fig. 7, has been also included in the natural periods analysis, confirming that 0.1 Hz resonances are indeed caused by OWC resonance as well, having the latter mode natural frequencies really similar to the former.

Given the large number of possible valve opening combinations and considering the primary objective of reducing rotational motions that contribute to displacements at the top of the tower, the focus of the study will be on the pitch and yaw motions. These two motions have exhibited the most significant improvements within the frequency ranges of [0 Hz; 0.09 Hz] and [0.12 Hz; 0.2 Hz], which highlights their potential for enhancing the performance of the hybrid system. In addition, surge motion has a significant impact in the displacement at the top of the tower, so it will be analyzed in a separate section, together with the bending moment at the tower base.

#### Platform stabilization, pitch and yaw motions

In this section, the experimental results are described, focusing on the effect of different configurations of OWCs on pitch and yaw motion stabilization. The response of the platform with each OWC combination is compared using the RAOs to the response when all OWC valves are closed, in order to detect local effects in certain intervals. Graphical representations are utilized, as depicted in Fig. 7, where as explained in Section “Design of the proposed hybrid six-floater oscillating water column-based floating offshore wind turbine platform concept”, the floater located in positive X-axis is named “1”, and the numbering of the remaining ones follows in a clockwise sense.

**Figure 8:**
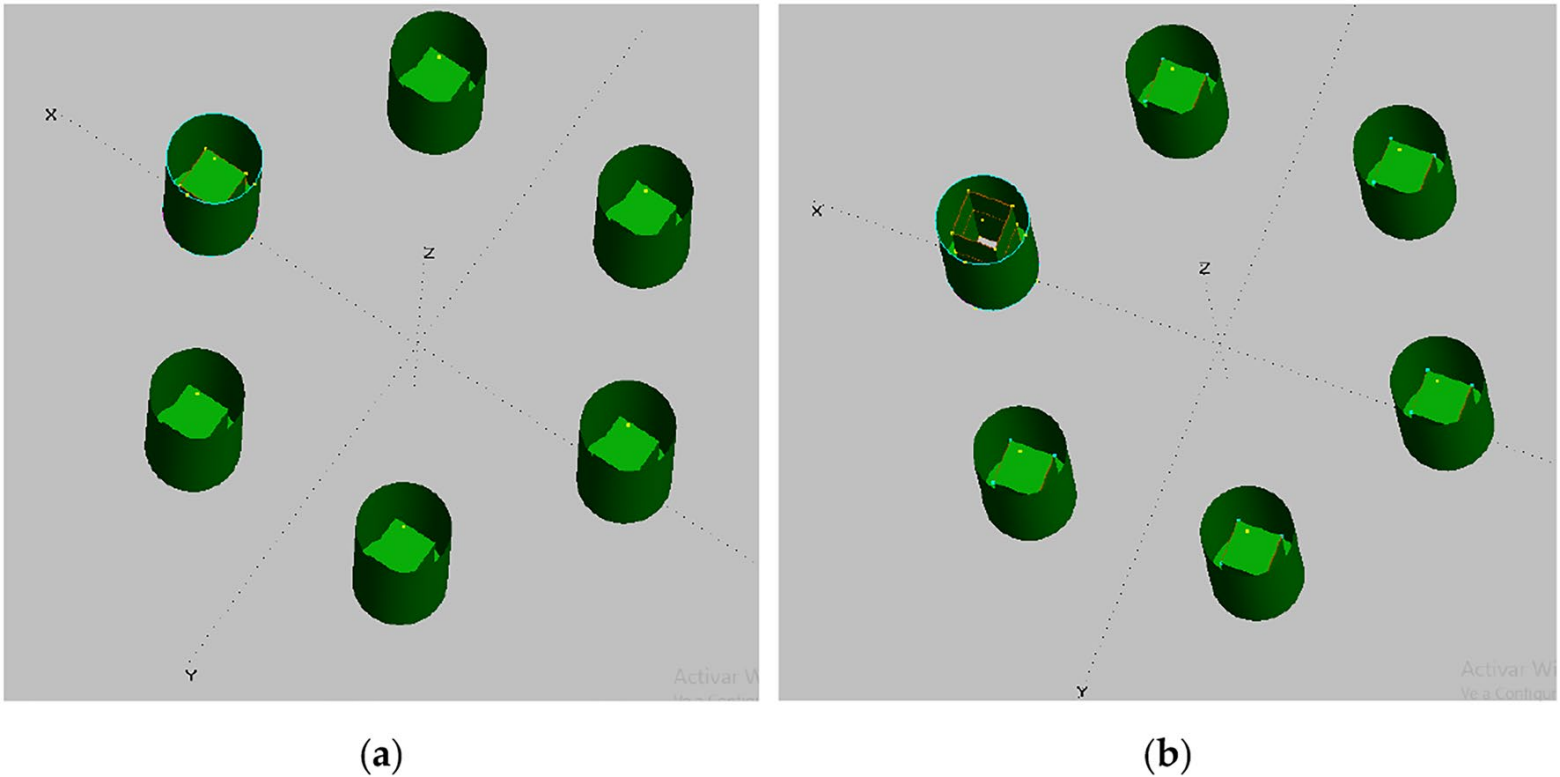
(**a**) Six-Floater platform model with all OWCs valves closed (code 000 000); (**b**) Six-Floater platform model with OWC valve in position 1 open (code 100 000).

The OWC operation mode is denoted by a six-digit code, where each digit represents the state of the corresponding OWC on the floater. The first digit corresponds to the OWC located on floater "1," the second digit represents the OWC on floater "2," and so on. The state of each OWC can be either "0" for a closed control valve or "1" for an open control valve. In Fig. 7, the RAO comparison is shown between the common "Mode 000 000," where all control valves are closed, and "Mode 100 000," where the control valve of the OWC on floater "1" is open while the remaining OWC control valves are closed. Figure 8 provides a graphical representation of this comparison for better understanding.

The analysis considers a total of sixty-three different combinations of open and closed chambers for the OWCs, with simulations under two different wind conditions. These models are computed to study the impact of the OWCs on the pitch and yaw motions of the platform. The behavior of the OWCs, with respect to their effect on these motions, can be classified into three groups: motion reduction, motion maintaining, and motion resonance, both locally, using their RAOs, and generally in the whole interval, using their PSD integral. The categorization for both pitch and yaw motions is discussed in the following sections.

##### Pitch motion reduction

**Figure 9:**
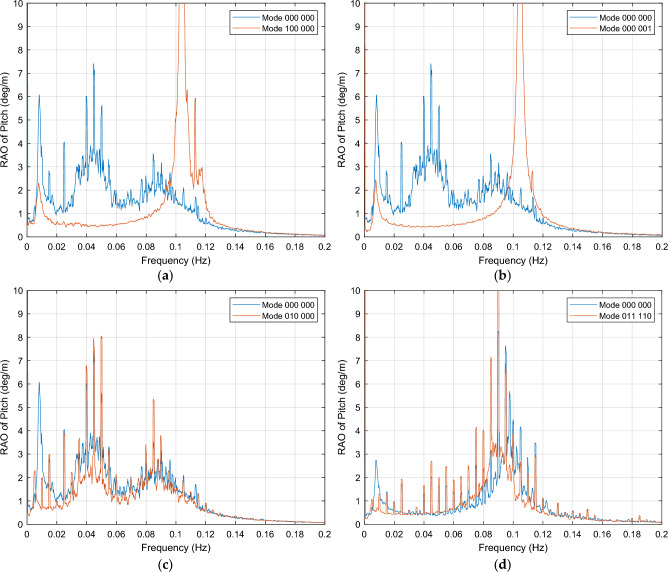
OWCs operation modes in which motion dampening occurs. (**a**) Mode 100 000, dampening within the 0.01 Hz to 0.08 Hz interval for near-rated wind speed; (**b**) Mode 000 001 for near-rated wind excitation, displaying an improved behavior within the 0.00 Hz to 0.09 Hz range, both in amplitude response and steady behavior, with a response signal clear of harmonics; (**c**) Mode 010 000, PSD integral reduction of -19.0% in general interval for near-rated wind condition; (**d**) Mode 011 110, for above-rated wind speed.

This section presents the combinations of OWCs that result in pitch motion reduction when determined valves of the OWC are open. For the near rated wind speed condition of 12 m/s, this group is characterized by a dampening response that occurs in the frequency range of 0.01 Hz to 0.09 Hz, followed by a resonance peak typically appearing in the interval of 0.10 Hz to 0.12 Hz. The behavior of these configurations is illustrated in Fig. 9 (a). To be classified as part of the pitch reduction group, the OWC combination should exhibit a resonance peak at a frequency greater than 0.08 Hz. This pitch reduction set can be subdivided into two groups: the first corresponds to those OWCs combinations that produce and increased response after the resonance peak, and the second group consists of those modes where there is no amplification after the peak. The second group is of particular interest due to its improved dampening response. For instance, Mode 000 001, as depicted in Fig. 9b, achieves a 50% reduction in response from 0.02 to 0.08 Hz, and a 80% reduction within the [0.03, 0.05] Hz interval. Thus, this mode enhances the platform's original response (with all OWCs out of operation) across all frequencies, except for the peak interval.

There is also another dampening behavior in the whole frequency interval, with no resonance peaks, as shown in Fig. 9c. The OWC configurations that lead to pitch dampening for the near rated wind speed condition are listed in Table 5.

**Table 5:** Effect of OWC combinations with wind excitation close to rated speed (12 m/s). PSD integral is calculated in the entire frequency interval [0.00, 0.20] Hz, and compared to the respective value of mode 000 000, baseline model. Local behavior is obtained through analysis of RAOs.

OWC Mode Code	Description: OWC(s) in operation, chamber number(s) with open valve	RAO pitch, 12 m/s. local behavior [interval] Hz	PSD integral Pitch, 12 m/s	RAO yaw, 12 m/s. Local behavior [interval] Hz	PSD integral yaw, 12 m/s	RAO surge, 12 m/s. Local behavior [interval] Hz	PSD integral Surge, 12 m/s	RAO Tower-base bending moment, 12 m/s, Local behav. [interval] Hz	PSD integral tower-base fore-aft bending moment, 12 m/s
000 000	All valves closed (for reference)		0.0%		0.0%		0.0%		0.0%
100 000	1	Damp. [0.01 − 0.08]	24.9%		19,855.9%		− 71.0%		2778.4%
010 000	2	Maint. [0.0 − 0.2]	− 19.0%	Damp. [0.0 − 0.2]	22.4%		− 57.2%		13.2%
001 000	3	Maint. [0.0 − 0.2]	16.7%	Damp. [0.0 − 0.2]	17.6%		− 55.8%		3.3%
000 100	4	Damp. [0.01 − 0.08]	− 4.7%		3661.8%		− 50.1%		2328.1%
000 010	5	Maint. [0.0 − 0.2]	0.7%	Damp. [0.0 − 0.2]	14.2%		− 57.0%		9.0%
000 001	6	Damp. [0.01 − 0.08]	− 45.0%	Damp. [0.0 − 0.2]	− 95.5%		− 76.1%		2791.3%
110 000	1, 2	Maint. [0.0 − 0.2]	25.6%	Damp. [0.0 − 0.2]	17.9%		− 56.7%		2.1%
101 000	1, 3	Damp. [0.01 − 0.08]	65.8%		52,505.3%		− 80.1%		69,094.4%
100 100	1, 4	Maint. [0.0 − 0.2]	45.8%	Maint. [0.0 − 0.2]	− 4.6%		− 0.5%		13.0%
100 010	1, 5	Maint. [0.0 − 0.2]	12.5%	Damp. [0.0 − 0.2]	17.6%		− 56.4%		1.0%
100 001	1, 6	Maint. [0.0 − 0.2]	22.2%	Damp. [0.0 − 0.2]	23.3%		− 56.3%		3.0%
011 000	2, 3	Maint. [0.0 − 0.2]	11.9%	Maint. [0.0 − 0.2]	15.6%		2.1%		10.1%
010 100	2, 4	Damp. [0.01 − 0.08]	147.2%		34,399.2%		− 73.7%		94,454.4%
010 010	2, 5	Maint. [0.0 − 0.2]	20.8%	Maint. [0.0 − 0.2]	− 3.2%		2.1%		− 0.6%
010 001	2, 6	Maint. [0.0 − 0.2]	25.5%	Maint. [0.0 − 0.2]	2.2%		2.8%		− 0.1%
001 100	3, 4	Maint. [0.0 − 0.2]	50.2%		3091.7%		− 58.6%		2250.9%
001 010	3, 5	Maint. [0.0 − 0.2]	7.9%	Maint. [0.0 − 0.2]	− 0.9%		2.3%		− 1.8%
001 001	3, 6	Maint. [0.0 − 0.2]	13.1%	Maint. [0.0 − 0.2]	2.2%		0.7%		− 0.5%
000 110	4, 5	Damp. [0.01 − 0.08]	442.4%		348,020.5%		− 59.4%		148,994.0%
000 101	4, 6	Damp. [0.01 − 0.08]	629.5%		522,868.4%		− 71.9%		133,151.7%
000 011	5, 6	Maint. [0.0 − 0.2]	15.0%	Maint. [0.0 − 0.2]	35.1%		1.0%		3.8%
111 000	1, 2, 3	Maint. [0.0 − 0.2]	72.5%	Damp. [0.0 − 0.2]	10.3%		− 55.6%		6.3%
110 100	1, 2, 4	Maint. [0.0 − 0.2]	67.2%	Damp. [0.0 − 0.2]	13.7%		− 55.5%		8.1%
110 010	1, 2, 5		7549.8%		31,212,752.7%		− 84.4%		137,681.6%
110 001	1, 2, 6	Maint. [0.0 − 0.2]	138.8%	Damp. [0.0 − 0.2]	26.2%		− 55.0%		1.7%
101 100	1, 3, 4	Maint. [0.0 − 0.2]	6.5%	Damp. [0.0 − 0.2]	13.4%		− 55.8%		1.1%
101 010	1, 3, 5	Maint. [0.0 − 0.2]	17.1%	Damp. [0.0 − 0.2]	18.4%		− 55.7%		4.8%
101 001	1, 3, 6	Maint. [0.0 − 0.2]	68.0%	Damp. [0.0 − 0.2]	30.3%		− 55.6%		− 0.4%
100 110	1, 4, 5	Maint. [0.0 − 0.2]	12.2%	Damp. [0.0 − 0.2]	20.9%		− 55.9%		− 0.4%
100 101	1, 4, 6	Maint. [0.0 − 0.2]	60.1%	Damp. [0.0 − 0.2]	25.9%		− 55.6%		− 1.7%
100 011	1, 5, 6	Maint. [0.0 − 0.2]	68.7%	Damp. [0.0 − 0.2]	37.4%		− 55.9%		9.0%
011 100	2, 3, 4	Maint. [0.0 − 0.2]	− 24.3%	Damp. [0.0 − 0.2]	5.1%		− 55.3%		5.6%
011 010	2, 3, 5	Damp. [0.01 − 0.08]	2884.8%		311,279.4%		− 86.4%		126,663.0%
011 001	2, 3, 6	Damp. [0.01 − 0.08]	2065.8%		282,150.9%		− 89.8%		79,919.6%
010 110	2, 4, 5	Maint. [0.0 − 0.2]	− 14.1%	Damp. [0.0 − 0.2]	12.6%		− 55.4%		0.7%
010 101	2, 4, 6		2295.2%		18,247,057.9%		− 88.6%		5282.0%
010 011	2, 5, 6	Maint. [0.0 − 0.2]	25.8%	Damp. [0.0 − 0.2]	27.4%		− 55.7%		2.5%
001 110	3, 4, 5		81.8%		396,252.1%		− 58.2%		733.2%
001 101	3, 4, 6	Maint. [0.0 − 0.2]	− 25.6%	Damp. [0.0 − 0.2]	18.5%		− 55.2%		4.4%
001 011	3, 5, 6	Maint. [0.0 − 0.2]	− 15.8%	Damp. [0.0 − 0.2]	24.1%		− 55.5%		− 0.4%
000 111	4, 5, 6	Maint. [0.0 − 0.2]	− 22.3%	Damp. [0.0 − 0.2]	26.9%		− 55.4%		− 0.1%
111 100	1, 2, 3, 4	Maint. [0.0 − 0.2]	51.6%	Damp. [0.0 − 0.2]	− 4.8%		− 54.4%		− 1.0%
111 010	1, 2, 3, 5	Maint. [0.0 − 0.2]	92.1%	Damp. [0.0 − 0.2]	8.4%		− 54.9%		1.0%
111 001	1, 2, 3, 6	Maint. [0.0 − 0.2]	211.2%	Damp. [0.0 − 0.2]	17.2%		− 54.9%		1.8%
110 110	1, 2, 4, 5	Maint. [0.0 − 0.2]	59.1%	Damp. [0.0 − 0.2]	16.2%		− 54.7%		2.0%
110 101	1, 2, 4, 6	Maint. [0.0 − 0.2]	184.6%	Damp. [0.0 − 0.2]	27.5%		− 54.6%		5.3%
110 011	1, 2, 5, 6	Maint. [0.0 − 0.2]	223.2%	Damp. [0.0 − 0.2]	44.6%		− 54.7%		3.0%
101 110	1, 3, 4, 5	Maint. [0.0 − 0.2]	− 33.7%	Damp. [0.0 − 0.2]	5.4%		− 54.6%		− 1.6%
101 101	1, 3, 4, 6	Maint. [0.0 − 0.2]	48.5%	Damp. [0.0 − 0.2]	27.5%		− 54.3%		− 100.0%
101 011	1 3, 5, 6	Maint. [0.0 − 0.2]	82.6%	Damp. [0.0 − 0.2]	36.5%		− 54.3%		2.8%
011 110	2, 3, 4, 5	Maint. [0.0 − 0.2]	− 61.4%	Damp. [0.0 − 0.2]	− 6.2%		− 54.1%		1.1%
011 101	2, 3, 4, 6	Maint. [0.0 − 0.2]	− 16.2%	Damp. [0.0 − 0.2]	0.8%		− 54.6%		2.0%
011 011	2, 3, 5, 6	Maint. [0.0 − 0.2]	14.9%	Damp. [0.0 − 0.2]	19.4%		− 54.2%		1.9%
010 111	2, 4, 5, 6	Maint. [0.0 − 0.2]	− 9.4%	Damp. [0.0 − 0.2]	26.5%		− 54.4%		− 0.4%
001 111	3, 4, 5, 6	Maint. [0.0 − 0.2]	− 60.9%	Damp. [0.0 − 0.2]	20.3%		− 54.1%		− 7.6%
111 110	1, 2, 3, 4, 5	Maint. [0.0 − 0.2]	− 4.8%	Damp. [0.0 − 0.2]	− 11.6%		− 53.3%		1.9%
111 101	1, 2, 3, 4, 6	Maint. [0.0 − 0.2]	160.4%	Damp. [0.0 − 0.2]	0.8%		− 53.5%		2.0%
111 011	1, 2, 3, 5, 6	Maint. [0.0 − 0.2]	252.1%	Damp. [0.0 − 0.2]	24.6%		− 53.2%		5.9%
110 111	1, 2, 4, 5, 6	Maint. [0.0 − 0.2]	165.8%	Damp. [0.0 − 0.2]	48.1%		− 54.0%		8.0%
101 111	1, 3, 4, 5, 6	Maint. [0.0 − 0.2]	− 6.3%	Damp. [0.0 − 0.2]	36.8%		− 53.2%		− 4.3%
011 111	2, 3, 4, 5, 6	Maint. [0.0 − 0.2]	− 62.5%	Damp. [0.0 − 0.2]	5.0%		− 52.5%		− 4.5%
111 111	1, 2, 3, 4, 5, 6		69,316.9%		354,773.8%		9.7%		30,987.2%

For the wind speed condition below the cut-out threshold of 24 m/s, the behavior is illustrated in Fig. 9d. Mode 011 110 provides a general motion reduction of -55.4% in the PSD integral in the entire interval. Both the response of the 000 000 mode and the mode under analysis change under the new wind condition, being the response patterns more similar within this group. The OWC configurations that lead to pitch dampening under the below cut-out wind speed condition are listed in Table 6. In this scenario, the PSD integral of pitch motion is a better tool to ascertain the effect of the different combinations, highlighting configurations that exhibit a dampening effect those with negative values of the PSD integral.

**Table 6:** Effect of OWC combinations with wind excitation below cut-out speed (24 m/s). PSD integral is calculated in the entire frequency interval [0.00, 0.20] Hz, and compared to the respective value of mode 000 000, baseline model. Local behavior is obtained through analysis of RAOs.

OWC Mode Code	Description: OWC(s) in operation, chamber number(s) with open valve	RAO pitch, 24 m/s. local behavior [interval] Hz	PSD integral pitch, 24 m/s	RAO yaw, 24 m/s. local behavior [interval] Hz	PSD integral Yaw, 24 m/s	RAO surge, 24 m/s. local behavior [interval] Hz	PSD integral Surge, 24 m/s	RAO tower-base bending moment, 24 m/s, local behav. [interval] Hz	PSD integral tower-base fore-aft bending moment, 24 m/s
000 000	All valves closed (for reference)		0.0%		0.0%		0.0%		0.0%
100 000	1		192.8%		− 46.2%		53.4%		− 50.7%
010 000	2		38.1%		− 87.6%		− 35.0%		− 0.7%
001 000	3		31.8%		16.6%		− 52.4%		2.8%
000 100	4		302.2%		185.9%		37.1%		92.8%
000 010	5		15.1%		8.9%		− 56.6%		1.2%
000 001	6		279.4%		907.9%		− 58.6%		4216.1%
110 000	1, 2		45.8%		16.5%		− 54.2%		7.2%
101 000	1, 3		296.3%		4491.1%		− 26.2%		165,168.8%
100 100	1, 4		85.9%		43.4%		− 8.3%		19.2%
100 010	1, 5		27.9%		11.4%		− 54.9%		2.5%
100 001	1, 6		33.3%		13.3%		− 55.0%		8.2%
011 000	2, 3		28.0%		13.3%		− 5.7%		8.3%
010 100	2, 4		178.2%		− 73.6%		10.3%		259,407.1%
010 010	2, 5		24.6%		3.1%		− 5.4%		12.4%
010 001	2, 6		33.3%		2.6%		− 5.7%		9.8%
001 100	3, 4		1727.3%		15,957.7%		− 53.2%		188,591.4%
001 010	3, 5	Damp. [0.09 − 0.2]	3.8%		2.5%		− 5.5%		7.3%
001 001	3, 6	Damp. [0.09 − 0.2]	3.8%		3.0%		− 5.6%		− 0.2%
000 110	4, 5		583.0%		27,768.8%		− 33.2%		171,719.8%
000 101	4, 6		1116.5%		50,218.0%		− 56.6%		286,438.2%
000 011	5, 6		33.7%		0.5%		− 5.6%		7.6%
111 000	1, 2, 3	Damp. [0.09 − 0.2]	89.9%	Damp. [0.02 − 0.2]	18.3%		− 52.4%		5.6%
110 100	1, 2, 4	Damp. [0.09 − 0.2]	95.9%	Damp. [0.02 − 0.2]	20.1%		− 52.7%		28.0%
110 010	1, 2, 5		13,900.0%		2,916,655.4%		− 71.2%		96,937.0%
110 001	1, 2, 6		9627.4%		1,988,955.7%		− 73.1%		18,792.8%
101 100	1, 3, 4	Damp. [0.09 − 0.2]	11.1%	Damp. [0.02 − 0.2]	16.1%		− 52.5%		3.0%
101 010	1, 3, 5		9491.7%		4,421,234.7%		− 86.3%		12,837.8%
101 001	1, 3, 6		8210.7%		2,794,563.4%		− 88.9%		20,485.3%
100 110	1, 4, 5	Damp. [0.09 − 0.2]	32.9%	Damp. [0.02 − 0.2]	15.9%		− 53.1%		7.1%
100 101	1, 4, 6	Damp. [0.09 − 0.2]	71.1%	Damp. [0.02 − 0.2]	21.4%		− 53.2%		5.1%
100 011	1, 5, 6		619.9%		44,103.3%		− 56.5%		879.9%
011 100	2, 3, 4	Damp. [0.09 − 0.2]	− 6.5%		10.0%		− 52.1%		6.2%
011 010	2, 3, 5		4433.1%		24,344.0%		− 80.3%		47,962.1%
011 001	2, 3, 6		2732.5%		27,622.0%		− 86.2%		36,082.1%
010 110	2, 4, 5	Damp. [0.09 − 0.2]	− 10.7%	Damp. [0.02 − 0.2]	14.7%		− 52.5%		7.9%
010 101	2, 4, 6		6753.1%		3,928,974.5%		− 87.8%		9418.2%
010 011	2, 5, 6	Damp. [0.09 − 0.2]	43.9%	Damp. [0.02 − 0.2]	15.7%		− 53.1%		11.8%
001 110	3, 4, 5	Damp. [0.09 − 0.2]	− 40.9%	Damp. [0.02 − 0.2]	9.9%		− 52.4%		3.6%
001 101	3, 4, 6	Damp. [0.09 − 0.2]	− 14.3%	Damp. [0.02 − 0.2]	12.7%		− 52.3%		− 2.6%
001 011	3, 5, 6	Damp. [0.09 − 0.2]	− 0.9%	Damp. [0.02 − 0.2]	16.2%		− 52.7%		− 3.3%
000 111	4, 5, 6	Damp. [0.09 − 0.2]	− 15.8%	Damp. [0.02 − 0.2]	16.7%		− 52.8%		2.5%
111 100	1, 2, 3, 4	Damp. [0.09 − 0.2]	61.7%	Damp. [0.02 − 0.2]	13.1%		− 50.5%		6.1%
111 010	1, 2, 3, 5	Damp. [0.09 − 0.2]	111.7%	Damp. [0.02 − 0.2]	18.0%		− 50.6%		15.6%
111 001	1, 2, 3, 6	Damp. [0.09 − 0.2]	272.4%	Damp. [0.02 − 0.2]	23.4%		− 51.2%		17.4%
110 110	1, 2, 4, 5	Damp. [0.09 − 0.2]	64.0%	Damp. [0.02 − 0.2]	21.2%		− 50.7%		13.0%
110 101	1, 2, 4, 6	Damp. [0.09 − 0.2]	225.5%	Damp. [0.02 − 0.2]	27.2%		− 51.0%		22.3%
110 011	1, 2, 5, 6	Damp. [0.09 − 0.2]	294.0%	Damp. [0.02 − 0.2]	32.8%		− 51.2%		7.2%
101 110	1, 3, 4, 5		− 33.6%	Damp. [0.02 − 0.2]	15.2%		− 50.5%		21.5%
101 101	1, 3, 4, 6		51.3%	Damp. [0.02 − 0.2]	20.3%		− 50.5%		8.5%
101 011	1 3, 5, 6		116.1%	Damp. [0.02 − 0.2]	24.5%		− 51.1%		19.8%
011 110	2, 3, 4, 5		− 55.4%	Damp. [0.02 − 0.2]	4.9%		− 50.0%		− 3.2%
011 101	2, 3, 4, 6		− 19.1%	Damp. [0.02 − 0.2]	8.8%		− 50.6%		6.0%
011 011	2, 3, 5, 6		27.7%	Damp. [0.02 − 0.2]	16.8%		− 50.5%		12.3%
010 111	2, 4, 5, 6		− 11.3%	Damp. [0.02 − 0.2]	19.6%		− 50.7%		14.0%
001 111	3, 4, 5, 6		− 57.7%	Damp. [0.02 − 0.2]	11.6%		− 50.3%		− 2.2%
111 110	1, 2, 3, 4, 5	Damp. [0.09 − 0.2]	11.0%	Damp. [0.02 − 0.2]	10.3%		− 47.9%		9.3%
111 101	1, 2, 3, 4, 6	Damp. [0.09 − 0.2]	188.0%	Damp. [0.02 − 0.2]	23.1%		− 48.5%		− 0.0%
111 011	1, 2, 3, 5, 6	Damp. [0.09 − 0.2]	333.0%	Damp. [0.02 − 0.2]	29.9%		− 49.0%		18.7%
110 111	1, 2, 4, 5, 6	Damp. [0.09 − 0.2]	216.7%	Damp. [0.02 − 0.2]	31.2%		− 49.5%		1.4%
101 111	1, 3, 4, 5, 6		− 9.5%	Damp. [0.02 − 0.2]	20.9%		− 48.8%		− 2.0%
011 111	2, 3, 4, 5, 6		− 45.0%	Damp. [0.02 − 0.2]	8.1%		− 47.7%		15.2%
111 111	1, 2, 3, 4, 5, 6		113,114.8%		67,855.7%		49.1%		51,811.1%

##### Pitch motion maintaining

This section presents those OWC combinations that exhibit platform responses where some of the OWCs are open, that are very similar to the response when all valves of the OWCs are closed.

**Figure 10:**
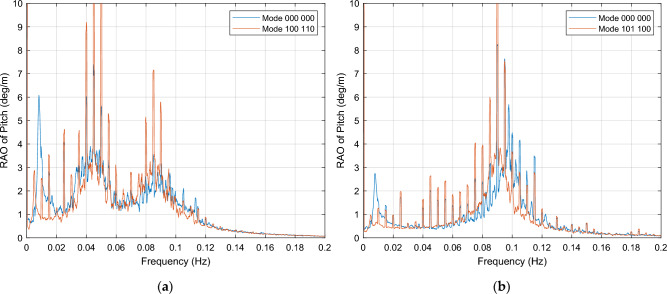
OWCs operation modes for pitch maintaining, with resonant peaks up to 0.09 Hz and negligible effect in [0.10, 0.20] Hz interval. (**a**) Mode 100 110, near rated wind speed condition of 12 m/s; (**b**) Mode 101 100, below cut-out wind speed condition of 24 m/s.

Regarding the near rated wind speed condition of 12 m/s, it is worth noting that most of these combinations generate resonant peaks in the same frequencies than mode 000 000. To avoid these resonant intervals, it is preferred that OWCs operate within frequencies where they have only a marginal effect on platform motion response, typically starting from 0.10 Hz. Table 5 provides a detailed description of these OWC combinations that produce a response with these characteristics. The effect of these combinations is compared to the response of the platform when all OWCs are out of operation (mode 000 000), as shown in Fig. 10 (a).

In Fig. 10 (a), it can be observed that the OWC operation modes for pitch maintaining exhibit resonant peaks up to 0.09 Hz, while having a negligible effect in the 0.10 to 0.20 Hz interval.

The response of the platform is generally maintained within the interval of [0.10, 0.20] Hz, with some frequencies within this range experiencing a slight dampening.

For wind speed below the cut-out threshold of 24 m/s, the effect cannot be determined through the RAO due to their similarity in shape to the motion reduction. Therefore, it is necessary to rely on the PSD integral of the pitch to conduct an adequate classification. The results of PSD integral calculations may be found in Table 6.

##### Pitch motion resonance

**Figure 11:**
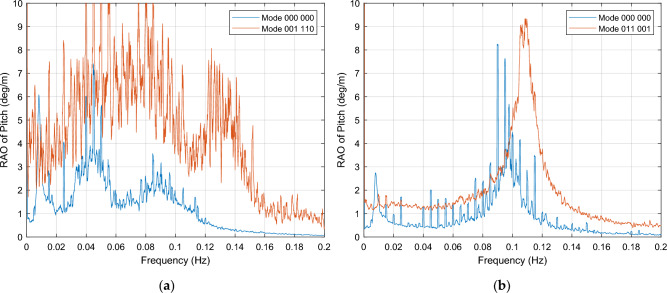
OWCs operation modes in which resonance of platform occurs. (**a**) Mode 001 110, for near rated wind speed condition; (**b**) Mode 011 001 for below cut-out wind speed condition.

This section considers the OWC associations that result in a resonance effect in pitch motion, for both cases under wind excitation. These combinations, described in Tables 5 and 6, and depicted in Fig. 11, should be avoided in order to achieve pitch motion reduction for platform stabilization. The behavior of these OWC operation modes is characterized by a resonance response across the entire [0.00, 0.20] Hz frequency interval.

##### Yaw motion reduction

**Figure 12:**
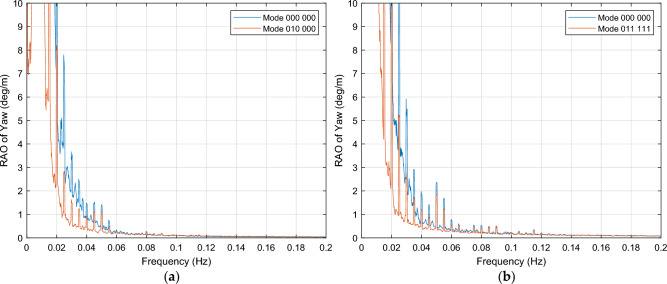
OWCs operation modes for yaw motion dampening. (**a**) Mode 010 000, for wind excitation close to rated speed; (**b**) Mode 011 111, for wind excitation close to cut-out speed.

**Figure 13:**
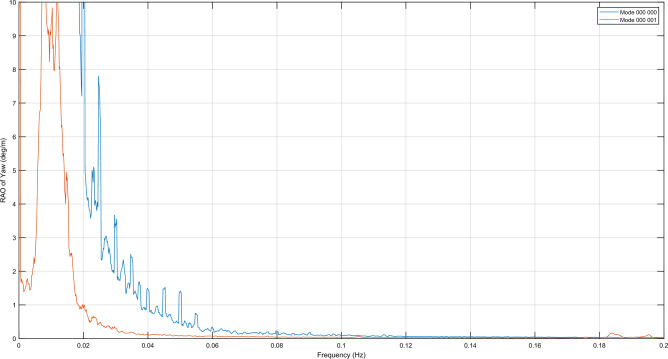
RAO of yaw motion for mode 000 001, in contrast with 000 000 mode. The PSD integral reveals an impressive motion reduction within all the frequency range of -95.5%, together with a starting of null yaw motion at 0.04 Hz.

This section presents those OWC combinations that result in a dampening effect on the yaw motion, for both wind load conditions considered in the simulations. The yaw motion is generally heavily dampened across the entire [0.00, 0.20] Hz frequency range, mainly for wind excitations close to the turbine rated wind speed, as depicted in Fig. 12. Upon analyzing the plots, it may be observed that regardless of the OWC combination, the incoming waves produce null or limited effect of on the yaw motion from 0.06 Hz onwards. Additionally, for wind condition close to rated speed, mode 000 001 stands out for shifting this null effect in the yaw motion from 0.06 to 0.04 Hz, and reduces the platform yaw motion by up to 50% compared to the response observed when all OWCs are inactive, as illustrated in Fig. 13.

Furthermore, this mode demonstrates a consistent and smoother response with less noise compared to mode 000 000. Besides, this mode also exhibits a positive impact on pitch motion reduction. These findings highlight the potential benefits of mode 000 001 for both pitch and yaw motion stabilization.

For both wind excitation cases, damping is observed from 0.02 Hz onwards. However, near the cut-out wind speed, the PSD integral of yaw motion for most of OWC combinations exhibits higher values across the entire [0.00, 0.20] Hz interval, indicating that resonance generally occurs within the [0.00, 0.02] Hz interval.

All OWC combinations that result in yaw motion reduction are listed in Tables 5 and 6.

##### Yaw motion maintaining

**Figure 14:**
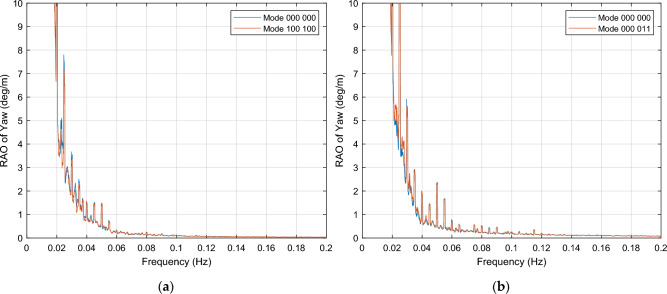
OWCs operation modes in which yaw motion maintaining occurs. (**a**) Mode 100 100, wind excitation close to rated speed; (**b**) Mode 000 011, wind excitation close to cut-out speed.

This section presents those OWC combinations that exhibit platform responses in yaw motion where some of the OWCs are open, that are very similar to the response when all valves of the OWCs are closed. The impact of some of these combinations is illustrated in Fig. 14, for both wind excitation cases. Tables 5 and 6 provide an overview of the OWC combination modes associated with this particular response. For wind speed below cut-out speed condition (24 m/s), a large number of OWC combinations show a PSD integral of yaw motion lower than 30% compared to the 000 000 baseline model value, across the entire frequency interval. The example selected in Fig. 14b is one of the few cases in which the difference with the baseline model is minimum (only + 0.5% in this case).

Upon analyzing the data, for wind condition close to rated speed, it may be observed that this response pattern occurs when OWCs operate in pairs. Additionally, it is important to emphasize that the yaw motion response is nearly identical to that of mode 000 000 in these cases, with only a small amplification observed in the [0.00, 0.02] Hz interval. This indicates that the presence of OWCs operating in pairs does not affect significantly the yaw motion response compared to the platform's baseline behavior.

##### Yaw motion resonance

**Figure 15:**
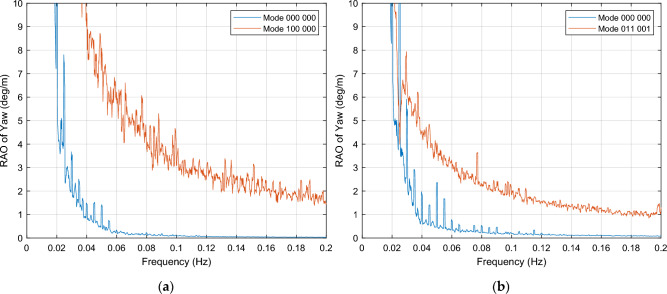
OWCs operation modes in which resonance occurs for yaw motion. (**a**) Mode 100 000, close to rated wind speed; (**b**) Mode 011 001, below cut-out wind speed.

This section considers the OWC associations that result in a resonance effect in yaw motion. An example of these combinations for every wind load case is displayed in Fig. 15 and all cases are listed in Tables 5 and 6. The response increment is extremely high in most cases, normally within the whole frequency interval.

#### Platform stabilization, other motions

In this section, a brief analysis of other motions not included in the general study is carried out. These other motions may have a significant impact when coupled with other DOFs or other induced actions.

##### Surge motion

Surge motion significantly affects the displacement at the top of the tower, being the aim of this article to minimize these displacements. The values of the PSD integral for surge motion under both wind excitations considered in this work, show that most OWC operation modes achieve an average reduction of 50%, as stated in Tables 5 and 6. This remarkable result further supports the 6OWC-FOWT platform as a reliable solution to improve the efficiency of the FOWT. Surge motion depends in turn in the mooring system, showing the layout used in this work successful results. Additional research will be necessary in order to assess the influence of mooring design in the 6OWC-FOWT platform concept.

##### Fore-aft bending moment at the tower base

This effect has been included in our study to provide valuable information when choosing which OWC combinations are more suitable for the 6OWC-FOWT platform operation. In certain scenarios, pitch motion may couple with fore-aft bending moment amplifying their effect. The results of PSD integral of fore-aft bending moment at tower based under different wind excitation conditions are shown in Tables 5 and 6.

## Discussion

The results obtained from our motion response analysis demonstrate that OWCs can effectively contribute to the stabilization of FOWT by selecting appropriate combinations of OWCs for specific wind conditions, sea states and frequency ranges of incident waves. Based on the effects observed in pitch and yaw motions resulting from various OWC combinations, and different wind excitation cases, the results can be classified into three groups:

- **Motion reduction** For wind excitations close to the wind turbine rated speed, this group shows a reduction in pitch motion within the [0.01, 0.08] Hz interval, indicating a dampening effect. Similarly, for yaw motion, this group exhibits a reduction in the entire [0.00, 0.20] Hz frequency range. Regarding wind excitations below the cut-out wind speed, it is necessary to rely on the integral of PSD of the selected motion to assess those cases in which its value is lower than the integral of PSD of the 000 000 baseline model.
- **Motion maintaining** In this group, for wind excitations close to the wind turbine rated speed, the pitch motion is maintained within the [0.10, 0.20] Hz interval, indicating minimal changes in response. Likewise, for yaw motion, the response remains consistent throughout the entire [0.00, 0.20] Hz frequency range. Similarly to the previous group, for wind excitations below the cut-out speed, the integral of PSD is to be used to ascertain those cases in which motion maintaining occurs.
- **Motion resonance** The motion resonance group experiences resonance effects in both pitch and yaw motions across the entire [0.00, 0.20] Hz frequency interval, for both wind excitation cases used in this study.

A summary of these motion responses is given in Table 7. These findings demonstrate the potential of OWCs in mitigating platform motions and the use of the integral of PDS as a metric, and RAOs for local study, provide valuable insights for selecting appropriate OWC combinations in different wind conditions, sea states and wave frequencies.

**Table 7:** Pitch and Yaw motion responses. For wind excitations close to the wind turbine rated speed, every motion group is related to the frequency interval in which it occurs, for certain OWCs combinations. For wind excitations below the cut-out wind speed, the motion group is categorized based on intervals of values provided by the integral of PSD. Mp, Rp, My and Ry are values to be defined.

Motion	Frequency interval (Hz) for rated wind speed	Integral of PSD for cut-out wind speed
Pitch reduction	[0.01, 0.08]	< 0
Pitch maintaining	[0.10, 0.20]	(0, Mp] (Mp: Maintaining pitch, to be defined)
Pitch resonance	[0.00, 0.20]	> Rp (Rp: Resonance pitch, to be defined)
Yaw reduction	[0.00, 0.20]	< 0
Yaw maintaining	[0.00, 0.20]	(0, My] (My: Maintaining yaw, to be defined)
Yaw resonance	[0.00, 0.20]	> Ry (Ry: Resonance yaw, to be defined)

In general, the effects of OWC combinations exhibit a mixture of motion reduction, motion maintaining, and motion resonance behaviors. The next step involves identifying the most suitable combination of OWCs for each wind condition and sea state, considering the frequency of the incoming waves. This selection process aims to dampen the pitch and yaw motions of the platform, thereby enhancing the overall efficiency of the floating offshore wind turbine.

Upon analyzing the results, an optimal OWC combination can be determined to ensure platform stabilization, based, as an example, on the following criterion: achieving maximum pitch and yaw motion dampening with the minimum number of OWCs. This criterion is driven by the goal of minimizing platform fabrication costs, future maintenance expenses, and maximizing energy generation for the selected OWC combination. Other figure of merit may be used to select a proper criterion in every case.

**Figure 16:**
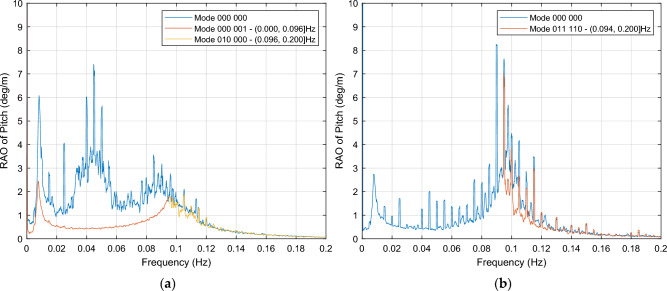
OCW selection for stabilization of pitch**.** (**a**) RAO of pitch motion response for mode 000 001, used in frequency interval (0.000, 0.096] Hz, and mode 010 000 used in frequency interval (0.096, 0.200] Hz, both compared to mode 000 000 for the entire interval, for wind excitations close to the wind turbine rated speed; (**b**) For wind excitations below the cut-out wind speed, mode 011 110 is selected for dampening the system from 0.094 Hz, mode 000 000 is to operate from 0 Hz to 0.094 locally because there is no other option.

In this way, considering wind excitations close to the wind turbine rated speed for the 6OWC-FOWT floating platform the OWC modes 000 001 and 010 000 are chosen for the pitch stabilization, as shown in Fig. 16. The mode 000 001 is to operate within the frequency range of [0.000, 0.096] Hz, and the mode 010 000 operates in [0.096, 0.200] Hz interval. From 0.09 Hz onward, the control system has to decide to choose between mode 010 000 and mode 000 000 (no OWCs action for the latter) to achieve the most effective reduction in pitch motion. In relation to wind excitations below the cut-out wind speed, it is necessary to rely on the PSD integral instead of RAOs to select the best case because the response of both the target OWC combination and the baseline platform are very similar in most cases, so it is not easy to find a local interval with the required effect, in such a case it exists.

The values of the PSD integral for every DOF of the different OWC combinations, in different wind conditions are calculated and shown in Tables 5 and 6, providing valuable information to select the best OWC combination, mainly used for winds above-rated speed, in which it is difficult to find a local interval with clear response of the OWC. Every integral of PSD of a determined DOF is accompanied by valuable information of local properties, as for example, local dampening or resonance, which is of help for decision making For instance, in order to select a suitable combination for our platform for above rated wind speeds, after searching the adequate table, four OWC candidates appear: 001 110, 011 110, 001 111 and 011 111. The four options have good response of pitch dampening, and although mode 001 111 has a better pitch reduction, mode 011 110 is chosen because it shows a better motion maintaining both for yaw motion and fore-aft moment at the tower base. Surge is reduced around 50% in all cases. Further investigation is needed about this surge effect and on finding a figure of merit to select an optimum OWC combination in every case.

Related to the mode 011 110, although it shows a good pitch reduction around 50% in the entire interval, it only provides local dampening in interval (0.094, 0,200] Hz, as it is displayed in Fig. 16b.

**Figure 17:**
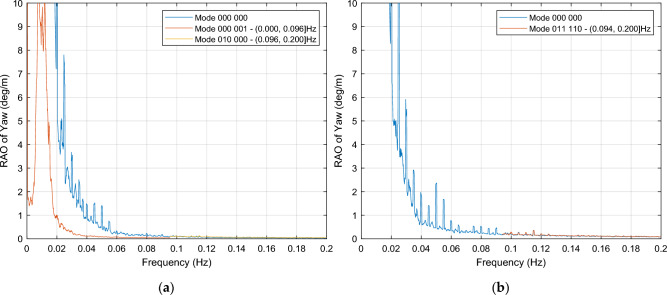
OCW selection for stabilization of yaw motion. (**a**) For near rated wind speeds, the yaw motion response of mode 000 001 is used in (0.000, 0.096] Hz, while mode 010 000 operates in interval (0.096, 0.200] Hz,; (**b**) Mode 011 110, for above rated wind speeds.

Figure 17b confirms that the selection of mode 011 110 has no effect on yaw motion within the previously stated interval.

Figure 16a illustrates a significant motion reduction in the frequency range of [0.000, 0.096] Hz for wind excitations close to the wind turbine rated speed. Furthermore, by implementing mode 010 000, a slight dampening effect is observed in specific frequencies within the [0.096, 0.120] Hz range. These OWC combinations, mode 000 001 and mode 010 000, not only contribute to pitch motion stabilization but also offer yaw motion stabilization within the same frequency intervals, showing a remarkable dampening within all the frequency range, as depicted in Fig. 17a. For above rated wind speeds, mode 011 110 is to operate for pitch reduction within the interval (0.094, 0.200] Hz, as illustrated in Fig. 16b, with no detrimental effect in yaw motion, as shown in Fig. 17b.

From Table 5 the values of the PSD integral for mode 000 001 can be obtained, showing a dampening in pitch of −45.0%, in yaw −95.5%, and eventually in surge a dampening of – 76.1%.

From the values of PSD integral displayed in Tables 5 and 6, yaw motion has a good maintaining effect, in both wind excitation cases, while surge shows an average reduction of −50% in both wind excitations for almost any OWC combination, so that it can be concluded that the 6OWC-FOWT platform is a promising potential solution to reduce the load in mooring lines and thus the cost related to mooring system. Further investigation is necessary in this area to properly assess the impact of this floating platform design and its applications.

The implementation of an advanced control system for this particular OWC operation mode in near rated wind excitation condition is straightforward, with a primary focus on pitch control within the (0.096, 0.200] Hz interval. Moreover, considering the favorable placement of the OWCs in the port bow and starboard bow areas for modes 000 001 and 010 000, respectively, they can function in tandem as "control fins," providing precise control over pitch and yaw motions based on the wave height. This is a promising research line that also involves the platform roll motion.

Although our research focuses on the OWC combinations that contribute to pitch motion reduction for platform stabilization, other combinations that belong to the pitch motion resonance group have the potential to expand the operational capabilities of the platform. These combinations can create operational modes that not only enhance the efficiency of the wind turbine but also increase the overall power extraction efficiency of the mixed platform with wind turbine and WECs when utilized as pure generators.

In light of this, the platform system can be operated in two distinct modes:

- **Wind mode** In this mode, the primary objective of OWC operation is the stabilization of the platform, aiming to reduce pitch motion and enhance the efficiency of the wind turbine.
- **Wave mode** In this mode, the wind turbine may be inactive due to environmental conditions or other factors, and the platform functions solely as a WEC. The OWCs can be utilized to bring the platform into resonance mode, thereby maximizing the harvesting of wave energy. It is important to note that in this approach, roll motion of the platform must also be taken into consideration. The selection criterion for OWC combinations may change, favoring the inclusion of a larger number of OWCs to optimize wave energy extraction.

Future research in platform stabilization could explore the expanded utilization of OWCs as WECs and the development of control strategies for collective OWCs control in both pitch and yaw stabilization scenarios. A key short-term objective would involve the formulation of control strategies for collective OWCs control specifically for pitch and yaw stabilization, employing the OWC modes of 000 001 and 010 000. The aim would be to determine the most appropriate OWC combination based on the frequency characteristics of the incident waves. Furthermore, the study of the floating platform aims to a comprehensive energy harvesting system, augmenting the role of OWCs beyond mere stabilizers and into exploiting potential as generators. This would imply developing corresponding control systems to optimize the overall energy harvesting capabilities of the platform, encompassing both wind and wave resources. Thus, the design and implementation of sophisticated control strategies can effectively coordinate the operation of multiple OWCs in a collective manner. These strategies should account for the varying environmental conditions and wave characteristics, enabling adaptive and efficient control of the OWCs for platform stabilization and energy extraction.

## Conclusions

This study presents a novel hybrid wind-wave floating offshore platform concept, known as the 6OWC-FOWT floater platform, which combines a wind turbine with WECs. The platform design features six floaters arranged around a 5 MW NREL wind turbine, with each floater housing an OWC chamber.

The design of the 6OWC-FOWT floater platform was developed using MultiSurf, and the hydrodynamic coefficients were computed using the WAMIT software. The resulting data from WAMIT were then integrated into FAST for further analysis. The OWC chambers were modeled to include controlled valves that allowed for the investigation of different opening/closing combinations and their effects on platform behavior. The primary objective of utilizing the OWCs in this study was to investigate their effectiveness as a stabilization system for the 6OWC-FOWT floater platform. By reducing the pitch and yaw motions, the platform's response is dampened, thereby improving the efficiency of the floating offshore wind turbine and extending its operational frequency range. Of particular interest, for near rated wind speed excitations, is the significant reduction in yaw motion achieved by the OWC combinations of mode 000 001 and mode 010 000, which exhibits a reduction four times greater than that observed in mode 000 000. Regarding above rated, close to cut-out speeds, the analysis is not straightforward and it is necessary a deeper analysis to find the most suitable combination. Last, the 6OWC-FOWT floater platform provides for the two wind excitation cases analyzed a surge motion reduction greater than 50% in most of OWC combination cases, which has a significant impact in the mooring system and its related costs, and needs further research to assess the extent of this finding.

The results indicate that the opening/closing combinations of the OWC chambers yield three distinct structural behaviors, namely motion reduction, motion maintaining, and motion resonance, depending on the frequency of the incident waves and wind excitation. By employing appropriate opening/closing combinations for motion reduction and motion maintaining, the OWCs can effectively function as stabilizers throughout the entire range of wave frequencies and wind conditions. This dampening effect enhances the platform's stability and improves the wind turbine's efficiency in wind harvesting mode.

The integration of OWCs as a stabilization system in the 6OWC-FOWT floater platform has yielded promising outcomes, such as motion reduction, improved turbine efficiency, and expanded operational frequency range. These findings underscore the potential of further research in this field, to advance the development of hybrid wind-wave floating offshore platforms and enhance their overall performance in renewable energy generation.

## Data Availability

The data used to support the findings of this study are available from the corresponding author upon request.
